# An arithmetic operation P system based on symmetric ternary system

**DOI:** 10.1371/journal.pone.0312778

**Published:** 2024-11-01

**Authors:** Hai Nan, Jie Zhang, Ping Guo, Jiqiao Jiang, Xu Zhang

**Affiliations:** 1 School of Computer Science and Engineering, Chongqing University of Technology, Chongqing, Chongqing, China; 2 School of Artificial Intelligence and Big Data, Chongqing Metropolitan College of Science and Technology, Chongqing, Chongqing, China; 3 School of Big Data and Computer Science Engineering, Chongqing College of Mobile Communication, Chongqing, Chongqing, China; GH Raisoni College of Engineering and Management Pune, INDIA

## Abstract

Nowadays, electronic computers use a “*binary*” numbering system, as opposed to “*ternary*” logic, which is closer to the way the human brain thinks. In this paper, the symmetric ternary system is applied to membrane computing for the first time. By combining the symmetric ternary system with membrane computing, this paper provides a more suitable arithmetic operation method for bio-computers, which breaks through the limitations of the traditional binary system in complex operations, and has a great potential for application in artificial intelligence and automatic learning in particular. The P System we designed include: Π^+^ for symmetric ternary addition, Π* for symmetric ternary multiplication, and Π^/^ for symmetric ternary division. The operation process of each P System was explained through examples, and their feasibility and effectiveness were verified through simulation software, UPSimulator. The system we designed can be further applied to symmetric ternary applications.

## 1 Introduction

Existing computers use a “binary” numbering system, which, despite the simplicity of its computational rules, is not a perfect representation of what humans really think. In contrast, “ternary” logic is much closer to the way the human brain thinks.

Ternary is the base 3 for the system, generally has two forms of expression: one is to “0”, “1”, “2” as the basic character form of expression. One is a representation with “-1”, “0”, “1” as the base character, and this representation is also known as symmetric or balanced ternary. In general, we do not have only “true” and “false” answers to questions, but also “I don’t know”. In symmetric triadic logic, the symbol “1” represents “true”; the symbol “-1” represents “false”; the symbol “0” represents “I don’t know”. Obviously, this logical expression is more in line with the development trend of computers in artificial intelligence, which provides the possibility of fuzzy arithmetic and autonomous learning for computers. The logic of symmetric ternary is usually applied to decision-making [1], such as voting with “yes”, “no”, or “abstain”; trading with “buy”, “sell”, or “wait-and-see”; double-entry bookkeeping reflects the thinking of symmetric ternary; SQL database system adopts three-valued logic, which is the application of symmetric ternary.

However, ternary logic is not a new emphasis. Ternary computers have long had a precedent in the history of computer development. As early as the 1950s and 1960s, a group of researchers at Moscow State University designed the first ternary computers in the history of mankind, “Сетунь” and “Сетунь 70”. The “Сетунь” computers used symmetric trigonometry instead of normal trigonometry [2]. Symmetric ternary logic circuits are not only faster and more reliable than binary logic circuits, but also require less equipment and power. One of the characteristics of symmetric ternary code is symmetry, i.e., the consistency of the opposite numbers, so that, unlike binary code, there is no concept of an “unsigned number”. As a result, the architecture of a ternary computer is much simpler, more stable, and more economical. The instruction system is also easier to read and very efficient. At the same time, symmetric ternary can represent integers more naturally than binary, with fewer integer digits of smaller absolute value (omitting the zero before the first non-zero digit). The numbers it records can express the full range of integers, and the introduction of “-1” eliminates the need for an extra minus sign for negative numbers. Its corresponding logic circuits are “negative voltage”, “zero voltage” and “positive voltage”.

As computer technology continues to advance, symmetric ternary logic has once again attracted the attention of the scientific community. As chips are made smaller and smaller, semiconductors are gradually moving closer to the realm of quantum. Difficult problems like quantum tunneling, where we might have to put in a very large amount of effort to possibly improve efficiency a little bit, might have to start opening up other paths. And ternary, right now, is being resurrected in forms other than electronic computers. The electronic computer has only two base states, on and off. But photonic computers, there are light intensity, wavelength, phase, propagation direction and polarization of five states. Professor Yi Jin of Shanghai University, starting from the basic principles of constructing computers and the basic characteristics of light, for the first time combined light intensity and polarization direction to represent the three-valued information, and utilized the spinning effect of liquid crystals and polarizers to realize the interconversion and migration of the three optical states, which put forward a brand-new theory of optical computers—Ternary Optical Computer (referred to as the TOC) [3]. In 2019, Chinese physicist Guangchan Guo and his team successfully completed the transmission of a ternary quantum signal called “qutrit”, which is the first successful ternary study by scientists in the quantum field [4].

Meanwhile, membrane computing has gradually become a popular research area in biocomputing. Membrane computing (also known as P System) is a new branch of natural computing, which is a new model of computation based on the abstraction of the structure and function of living cells and the collaboration of cell population such as tissues and organs. It is a computational model proposed by the Professor Gh.Păun in 1998 [5]. After Gh.Păun published his paper “Computing with membranes” in 2000 [6], it marked the birth of membrane computing as a research field. Since its introduction, membrane computing has attracted extensive attention from the scientific community, covering a wide range of disciplines or fields such as computer graphics and linguistics [7], biology [8], automation [9], and economics [10], and has rapidly evolved into a field of scientific research with great potential, and its development provides a rich computational framework for bio-computing.

With the intensive research on membrane computing, several studies have been devoted to the development and optimization of P System for arithmetic operations. Adrian Atanasiu designed arithmetic cell-like P System [11]. G.Ciobanu [12] designed arithmetic P System based on natural coding to realize arithmetic operations, which greatly simplified the membrane system structure. Ping Guo et al. [13] designed multi-layer membrane P System to realize arithmetic operations and reduce the computational complexity. Haiyan Zhang et al. [14] designed a single-layer membrane P System to realize arithmetic operations, which further simplifies the membrane structure and improves the computational efficiency. Minghong Luo et al. [15] designed a multi-layer membrane P System to realize arithmetic operations with signed numbers. Ping Guo et al. [16–18] designed single-layer membrane P System to realize expression evaluation in the integer domain. Hong Zhang et al. [19] implemented basic arithmetic operations in the domain of rational numbers using P System. Kong, Y. et al. [20] investigated fundamental problems in fraction representation and arithmetic-fraction simplification. However all the above studies are based on binary or decimal.

While most research is still focused on binary and decimal systems, the potential of ternary is gradually emerging.Symmetric ternary is used in a number of applications due to its unique properties. Inspired by the balanced-ternary concept, Ji L et al. [21] demonstrates the reconfigurable generation of order-controllable vortices via cascaded N-layer meta surfaces. Faghih E et al. [22], for the first time, considers balanced ternary advantages to achieve a more efficient design for quantum multipliers as the main component in arithmetic blocks.

There are also many scientists who have devised arithmetic operations related to symmetric ternary. Ratan Kumar et al. [23] designs ternary logic circuits for nanoelectronics applications, the digital multiplier circuit is developed using Pseudo n-type carbon nanotube field effect transistors (CNTFETs). Based on the parallel carry-free TW-MSD adder, Yunfu S et al. [24] proposed a parallel R4-MSD square root algorithm, which is designed and implemented on the protype SD16 of ternary optical computer. Malik A et al. [25] proposes carbon nanotube field effect transistor (CNTFET)-based ‘exact’ and ‘approximate’ ternary full adders (TFA). Vudadha C [26] presents a new methodology to implement ternary Conditional Sum Adders (CSA) using CNFETs.

Although ternary has shown its potential for applications in several fields, its combination with membrane computing is still under-explored. And balanced ternary may become the most suitable number system for bio-computers. The study of arithmetic P System based on symmetric triples for membrane computing is of great academic and practical importance for the realization of a general-purpose bio-computer. The innovations of this paper mainly include:

1) Applying the symmetric ternary number system to membrane computing and designing a symmetric ternary arithmetic operation cell-like P System.
2) Dynamically creating cell membranes to realize arbitrary digit ternary arithmetic operations.
3) The symmetric ternary arithmetic operation system designed in this paper is simulated in UPSimulator (UPS), which is a simulator proposed in [27]. And the idea and feasibility of the algorithm are verified by examples.

The rest of the paper is organized as follows, Section 2 introduces the biological basis of membrane computing, describes the definition of cell-like P System. And then briefly introduces symmetric ternary and its arithmetic rules. Section 3 designs and implements an arithmetic P System based on symmetric ternary and detailing the rule execution process. Section 4 gives examples to elaborate the execution flow of the rule and verifies the correctness of the rule design through experimental simulation on computer. Section 5 summarizes the work accomplished in this paper and presents issues for future refinement.

## 2 Research foundation

### 2.1 Cell-like P system

**Fig 1:**
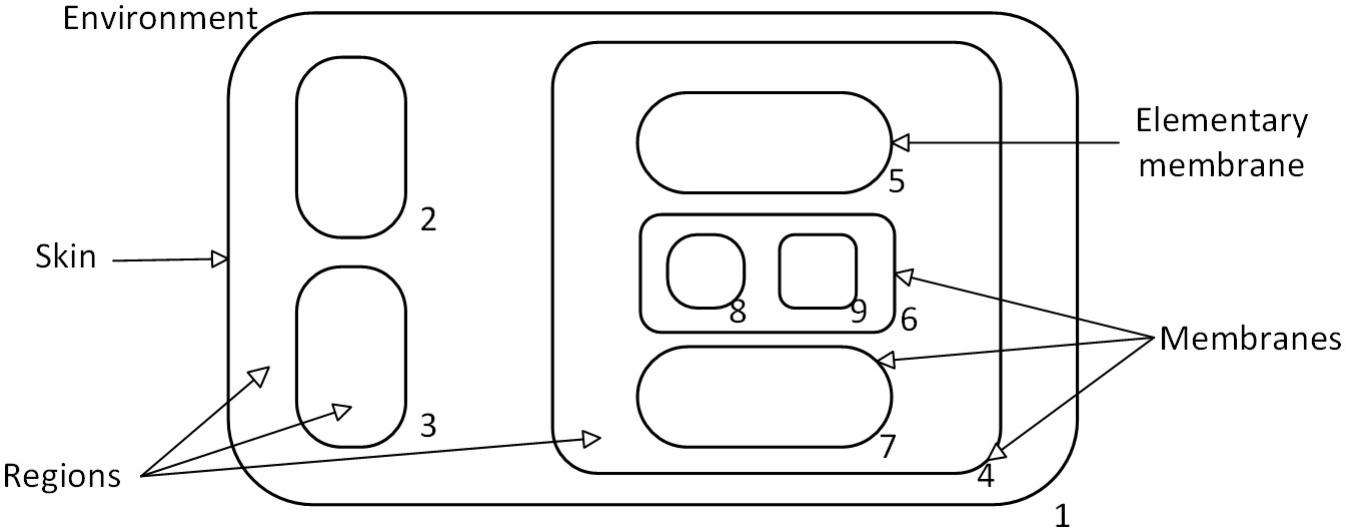
The structure of cell-like P system [6].

The cell-like P System is one of the most basic and earliest proposed model of membrane computing [6], and an abstract schematic of the cell-like P System is shown in Fig 1. The membrane computing model divides a cell into multiple regions with a hierarchical structure, and the boundary of each region is the membrane. The outermost membrane, called the skin, separates the entire membrane system from its external environment, and the region outside the skin is the environment. If there are no other membranes within the membrane, it is called the basic membrane [28]. Each membrane represents a region; the region of a basic membrane is the space it contains; the region of a non-basic membrane refers to the space between the membrane itself and the membrane it directly contains. Regions contain objects represented by multi-sets, and objects evolve by executing reaction rules: objects are converted into other objects that can reach a certain membrane, which can also be dissolved or split. The execution of rules follows a nondeterministic and parallel character. The time when there are no rules to be executed in the region is called downtime, and the results of the computation are sent in the specified membrane or environment.

Membrane structures can be represented by generalized tables. A membrane is denoted by a pair of brackets ‘[]’, with the subscripts of the brackets denoting the label of the membrane. The basic membrane i is denoted as [_*i*_]_*i*_; if membrane i contains membrane k inside, the membrane structure is denoted as [_*i*_[_*k*_]_*k*_]_*i*_. The membrane structure of Fig 1 can be represented by the generalized table [1 [2]_2_ [3]_3_ [4 [5]_5_ [6 [8]_8_ [9]_9_]_6_ [7]_7_]_4_]_1_.

A cell-like P System of degree m (*m* ≥ 1) is defined as formula (1) [6].

$$\begin{array}{c} \Pi =(V,\mu ,\omega_{1},\ldots ,\omega_{m},R_{1},\ldots ,R_{m},\rho_{1},\rho_{2},\ldots ,\rho_{m},i_{0}) \end{array}$$ (1)

where:

1. *V* is a finite non-empty alphabet, whose elements are objects;
2. *μ* is a membrane structure containing *m* membranes, where *m* is called the degree of Π;
3. *ω*_*i*_ ∈ V* (1 ≤ *i* ≤ *m*), denotes the multiset of objects contained inside region *i* in the membrane structure *μ*. V* is the set of arbitrary strings consisting of characters in *V*;
4. *R*_*i*_ (1 ≤ *i* ≤ *m*) is a finite set of evolutionary rules inside region *i* in the membrane structure *μ*, the evolutionary rules are binary groups (*u*, *v*), usually written as *u* → *v*, where, *u* is a string in *V*^+^ and *V*^+^ is a set of non-empty strings in V*, *v* = *v*′ or *v* = *v*′*δ*, here *v*′ is a string on the set $\left\{a_{\text{here}},a_{\text{out}},a_{{\text{in}}_{j}}|a\in V,1\leq j\leq m\right\}$, *δ* is a special character that does not belong to *V*, when a rule contains *δ*, the membrane is dissolved after executing the rule, the length of *u* is known as the radius of the rule *u* → *v*;
5. *ρ*_*i*_ (1 ≤ *i* ≤ *m*), denotes a partial order relation in *R*_*i*_;
6. *i*_*o*_ is a number between 1 and *m* where the output of results in Π.

In this paper, the initial grid refers to the P System that has not yet started the computation. When operands are sent into the P System, which triggers the rules to be executed, the computation starts. The P System at a certain time slice in the computation is called the configuration at that moment. As rules are executed, the configuration of the P System will change until there are no rules left to be executed.

In every membrane structure, the rules will be enforced according to the following two principles:

1) Uncertainty. The P System will follow the principle of uncertainty when executing rules, which means that when there are n evolutionary rules in the membrane that can be executed at the same time, the P System randomly selects some of the rules to be implemented and the objects in the system to be evolved and chooses the rule that governs this evolution in a non-deterministic way [28].
2) Maximum Parallelism. In the P System, each step of the computation follows the principle of maximum parallelism, which means that all the rules that can be executed must be executed at the same time.

### 2.2 Symmetric ternary

The symmetric ternary was inspired by Gauss’s idea of the simplest set of weights. The simplest set of weights problem is as follows: How should the simplest set of weights be designed for weighing an object of any integer gram weight with weights on a balance. Usually when weighing an object with weights on a balance, the object to be weighed is placed on one side of the balance pan and the weights on the other side. Gauss proposed that the simplest set of weights is 1, 3, 9, 27, …, 3^*n*^, … grams, and that the weights can be placed on either side of the balance pan when weighing an object. It can be shown that the formula for weighing an object of any integer gram weight with the simplest set of weights is expressed as follows [29]:

$$\begin{array}{c} K=a_{n}3^{n}+a_{n-1}3^{n-1}+\cdots +a_{1}3^{1}+a_{0}3^{0}. \end{array}$$ (2)

Where K is any positive integer, 3^*n*^, 3^*n*−1^, …, 3^1^, 3^0^ is the weight of each weight, the coefficients *a*_*n*_, *a*_*n*−1_, …, *a*_1_, *a*_0_ is one of −1,0,1. “1” represents that the weight is placed on the other side of the balance pan of the object to be weighed, “−1” represents that the weight is placed on the same side of the object to be weighed. And “0” means that the weight does not participate in the weighing. The 3^*n*^, 3^*n*−1^, …, 3^1^, 3^0^ are used to represent the mass of the weights are viewed as bit-weights, and ignoring the bit-weights, any positive integer *K* can be expressed in the following form [29]:

$$\begin{array}{c} K=a_{n},a_{n-1},\ldots ,a_{1},a_{0}. \end{array}$$ (3)

Where *a*_*n*_, *a*_*n*−1_, …, *a*_1_, *a*_0_ is one of −1,0,1. To avoid confusion, −1 is generally denoted by *T*, and *Z* or *z* under special conditions. In this paper, we denote −1 by *T*. This representation of an arbitrary integer *K* by a string of numbers consisting of the various coefficients is called symmetric ternary.

Symmetric ternary system has many advantages, first of all, it has both positive and negative number elements, which can be expressed as positive or negative numbers by the same Eq (2). The sign of the first digit can be used to determine whether *K* is positive or negative; i.e., when the first digit is positive, *K* is positive, and when the first digit is negative, *K* is negative. The quadratic operations for symmetric ternary are also simple, and Tables 1–4 shows the quadratic rules for symmetric ternary.

**Table 1:** Addition in symmetric ternary.

+	TT	T0	T1	T	0	1	1T	10	11
11	0	1	1T	10	11	1TT	10T	1T1	10T
10	T	0	1	1T	10	11	1TT	1T0	-
1T	T1	T	0	1	1T	10	11	-	-
1	T0	T1	T	0	1	1T	-	-	-
0	TT	T0	T1	T	0	1	-	-	-
T	T11	TT	T0	T1	T	0	-	-	-
T1	T10	T11	TT	-	-	-	-	-	-
T0	T1T	T10	-	-	-	-	-	-	-
TT	T01	-	-	-	-	-	-	-	-

**Table 2:** Subtraction in symmetric ternary.

-	TT	T0	T1	T	0	1	1T	10	11
TT	0	T	T1	T0	TT	T11	T10	T1T	T01
T0	1	0	T	T1	T0	TT	T11	T10	T1T
T1	1T	1	0	T	T1	T0	TT	T11	T10
1	10	1T	1	0	T	T1	T0	TT	T11
0	11	10	1T	1	0	T	T1	T0	TT
T	1TT	11	10	1T	1	0	T	T1	T0
T1	1T0	1TT	11	10	1T	1	0	T	T1
T0	1T1	1T0	1TT	11	10	1T	1	0	T
TT	I0T	1T1	1T0	1TT	11	10	1T	1	0

Note: Left column minus top row

**Table 3:** Multiplication in symmetric ternary.

*	TT	T0	T1	T	0	1	1T	10	11
11	T11T	TT0	T10	TT	0	11	10T	110	1TT1
10	TT0	T00	T10	T0	0	10	1T0	100	-
1T	T01	T10	1T	1	0	1T	11	-	-
1	TT	T0	T1	T	0	1	-	-	-
0	0	0	0	0	0	0	-	-	-
T	11	10	1T	1	0	T	-	-	-
T1	10T	1T0	11	-	-	-	-	-	-
T0	110	100	-	-	-	-	-	-	-
TT	1TT1	-	-	-	-	-	-	-	-

**Table 4:** Division in symmetric ternary.

/	TT	T0	T1	T	0	1	1T	10	11
TT	1	1.1	1T	11	-∞	TT	T1	T.T	T
T0	$1.\bar{\text{T1}}$	1	$1.\bar{1}$	10	-∞	T0	$\text{T}.\bar{\text{T}}$	T	$\text{T}.\bar{\text{1T}}$
T1	$1.\bar{\text{T}}$	1.T	1	1T	-∞	T1	T	T.1	$\text{T}.\bar{1}$
T	$0.\bar{\text{1T}}$	0.1	$0.\bar{1}$	1	-∞	T	$0.\bar{\text{T}}$	0.T	$0.\bar{\text{T1}}$
0	0	0	0	0	NaN	0	0	0	0
1	$0.\bar{\text{T1}}$	0.T	$0.\bar{\text{T}}$	T	+∞	1	$0.\bar{1}$	0.1	$0.\bar{\text{1T}}$
1T	$\text{T}.\bar{1}$	T.1	T	T1	+∞	1T	1	1.T	$1.\bar{\text{T}}$
10	$\text{T}.\bar{\text{1T}}$	T	$\text{T}.\bar{\text{T}}$	T0	+∞	10	$1.\bar{1}$	1	$1.\bar{\text{T1}}$
11	T	T.T	T1	TT	+∞	11	1T	1.1	

Note: Left column divided top row

## 3 Arithmetic P system based on symmetric ternary system

### 3.1 Addition and subtraction

According to the definition of cell-like P System, an addition and subtraction arithmetic P System based on symmetrical ternary can be defined as:

$$\begin{array}{c} \Pi^{+}=\left(V,\mu ,\omega_{1},\omega_{M_{1}},R,\rho ,i_{0}\right) \end{array}$$ (4)

Where:

*V* = {*a*, *b*, *c*, *T*, 0, 1, *s*, *f*, *E*, *Y*};

$\mu ={[}_{1}{[}_{M_{1}}{]}_{M_{1}}{]}_{1}$ ;

*ω*_1_ = {*Y*};

$\omega_{M_{1}}=\{f\}$ ;

*i*_0_ consists of *M*_1_ and his submembrane to hold the output;

*ρ* = 1, 2;

$R=R_{1}\cup R_{M_{1}}$ ;

*R*_1_ = {*r*_1_: (*T* → (*a*, *M*_1_), 1), *r*_2_: (0 → (*b*, *M*_1_), 1), *r*_3_: (1 → (*c*, *M*_1_), 1),

*r*_4_: (*sY* → *E*, 1), *r*_5_: (*E* → (*E*, *M*_1_), 1)};

$R_{M_{1}}=\{r_{1}:(af\to T[f],1),r_{2}:(bf\to 0[f],1),r_{3}:(cf\to 1[f],1),$

*r*_4_: (*Ef* → *E*[*f*], 1), *r*_5_: (*a* → (*a*, in), 2), *r*_6_: (*b* → (*b*, in), 2), *r*_7_: (*c* → (*c*, in), 2),

*r*_8_: (*aE* → *T*(*E*, in), 1), *r*_9_: (*bE* → 0(*E*, in), 1), *r*_10_: (*cE* → 1(*E*, in), 1),

*r*_11_: (0^2^ → 0, 1), *r*_12_: (0*T* → *T*, 1), *r*_13_: (01 → 1, 1), *r*_14_: (*T*^2^ → 1(*T*, in), 1),

*r*_15_: (*T*1 → 0, 1), *r*_16_: (1^2^ → *T*(1, in), 1)};

In this case, the correspondence of objects is as follows. The augend numbers *T*, 0, 1 are represented as objects *a*, *b*, *c* when they enter membrane *M*_1_ from membrane 1. Objects *a*, *b*, *c* reach to the membrane where object *f* is located, and are converted to objects *T*, 0 and 1 after reacting with *f*. In this way, the incoming objects *a*, *b*, and *c* will only react if they enter the membrane where *f* is located, thus enabling dynamic modeling and the storage of the augend numbers from low to high in membrane *M*_1_ and its submembranes. Object *s* is used to indicate the end of the augend input and to generate *E* with *Y* in Membrane 1. The purpose of *E* is twofold: to signal that the system is ready to input the addend, and to convert the addend to *T*, 0, and 1 so that it can be added to the augend.

Let us assume that the augend is *a*_*n*_*a*_*n*−1_…*a*_1_ and the addend is *b*_*m*_*b*_*m*−1_…*b*_1_. Then we illustrate the use of the rules in Π^+^ by adding these two numbers.

**(1) Input of the augend**:

We input one bit of the augend every two time slices from low to high. First *a*_1_ is sent into membrane 1.

- **Case 1**: *a*_1_ = *T*. Executing rule *r*_1_ in *R*_1_, object *T* is converted to object *a* and sent into membrane *M*_1_. Executing rule *r*_1_ in *R*_*M*_, object *a* and object *f* are consumed with *T* produced, a new membrane *M*_2_ is created at the same time, and *f* is sent into membrane *M*_2_.
- **Case 2**: *a*_1_ = 0. Executing rule *r*_2_ in *R*_1_, object 0 is converted to object *b* and sent into membrane *M*_1_. Executing rule *r*_2_ in *R*_*M*_, object *b* and object *f* are consumed with 0 produced, a new membrane *M*_2_ is created, and *f* is sent into membrane *M*_2_.
- **Case 3**: *a*_1_ = 1. Executing rule *r*_3_ in *R*_1_, object 1 is converted to object *c* and is sent into membrane *M*_1_. Executing rule *r*_3_ in *R*_*M*_, object *c* and object *f* are consumed with 1 produced, a new membrane *M*_2_ is created, and *f* is sent into membrane *M*_2_.

Object *a*_*i*_ (1 < *i* < *n*) is sent into membrane 1, converted to object *a*, *b*, or *c* (*R*_1_: *r*_1_ ∼ *r*_3_), and enters membrane *M*_1_, then continues into the inner membrane (*R*_*M*_: *r*_5_ ∼ *r*_7_) until it reaches membrane *M*_*i*_ where it is consumed with *f* and produces object *T*, 0, or 1 (*R*_*M*_: *r*_1_ ∼ *r*_3_).

The last object *a*_*n*_ is input to the system with *s*. Object *s* is used to indicate that the augend has been fully entered. Rule *r*_4_ in *R*_1_ is executed, object *s* is consumed with *Y* and *E* produced, indicating that the system is ready to input the addend, while *E* enters Membrane *M*_1_.

**(2) Input of the addend**:

Object *b*_1_ is sent into membrane 1.

- **Case 1**: *b*_1_ = *T*, rule *r*_1_ in *R*_1_ is executed, object *T* is converted to *a* to enter membrane *M*_1_. Object *a* is consumed with *E* (*R*_*M*_: *r*_8_), *a* is converted to *T* to be preserved in membrane *M*_1_, and *E* enters the inner membrane *M*_2_.
- **Case 2**: *b*_1_ = 0, executing rule *r*_2_ in *R*_1_. Object 0 is converted to *b* and is sent into membrane *M*_1_. Object *b* and *E* are consumed (*R*_*M*_: *r*_9_), generating 0 to remain in membrane *M*_1_ and *E* is sent into membrane *M*_2_.
- **Case 3**: *b*_1_ = 1, executing rule *r*_3_ in *R*_1_. Object 1 is converted to *c* to enter *M*_1_, then object *c* is consumed with *E* (*R*_*M*_: *r*_10_) and object 1 produced to stay in membrane *M*_1_, and *E* enters membrane *M*_2_.

The next object *b*_*i*_ (1 < *i* ≤ *m*) follows a similar pattern to *b*_1_. It is sent into membrane 1 first, and then converted to object *a* or *b* or *c* (*R*_1_: *r*_1_ ∼ *r*_3_), entering membrane *M*_1_. Object *b*_*i*_ continues into the inner membrane (*R*_*M*_: *r*_5_ ∼ *r*_7_) until it reaches membrane *M*_*i*_. Then it is consumed with *E* to produce object *T*, 0 or 1 (*R*_*M*_: *r*_1_ ∼ *r*_3_).

**(3) Addition**:

The process of addition is simultaneous with the input of the addend. When *b*_1_ reaches membrane *M*_1_, the addition operation can be performed without having to wait for the addend to be fully input. Two numbers are added bit-wise from low to high and may produce carrying. The possibilities of *a*_*i*_ + *b*_*i*_ (1 ≤ *i* ≤ min{*n*, *m*}) in *a*_*n*_*a*_*n*−1_…*a*_1_ and *b*_*m*_*b*_*m*−1_…*b*_1_ are as follows:

- **Case 1**: 0 + 0 = 0 (*R*_*M*_: *r*_11_);
- **Case 2**: 0 + *T* = *T* (*R*_*M*_: *r*_12_);
- **Case 3**: 0 + 1 = 1 (*R*_*M*_: *r*_13_);
- **Case 4**: *T* + *T* = *T*1 (*R*_*M*_: *r*_14_);
- **Case 5**: *T* + 1 = 0 (*R*_*M*_: *r*_15_);
- **Case 6**: 1 + 1 = 1*T* (*R*_*M*_: *r*_16_).

For Case 4 and Case 6 that have generated feeds, the high bit of the result is sent into the inner membrane and the low bit is left in the original membrane.

The result of the addition is saved in the membrane *M*_1_, …, *M*_*k*_ (*k* ≥ 1) from low to high. The example of addition in Section 4.1.1 provides a more concrete demonstration of the implementation of the rules. In the addition P System Π^+^, symmetric ternary numbers “*n* + *m*” require at most 3*n* + 4*m* time slices for addition.

The subtraction P System simply changes the rule *r*_1_ in *R*_1_ to (*T* → (*c*, *M*_1_)), and *r*_3_ to (1 → (*a*, *M*_1_)), the operations are all consistent with addition, so we won’t go into too much detail here.

### 3.2 Multiplication

According to the definition of cell-like P System, a multiplication arithmetic P System based on symmetrical ternary can be defined as:

$$\begin{array}{c} \Pi^{*}=\left(V,\mu ,\omega_{1},\omega_{M_{1}},R,\rho ,i_{0}\right) \end{array}$$ (5)

Where:

*V* = {*a*, *b*, *c*, *T*, 0, 1, *A*, *B*, *C*, *x*, *y*, *z*, *p*, *q*, *r*, *u*, *n*, *s*, *f*, *E*, *Y*};

$\mu ={[}_{1}{[}_{M_{1}}{]}_{M_{1}}{]}_{1}$ ;

*ω*_1_ = {*Y*};

$\omega_{M_{1}}=\{f\}$ ;

*i*_0_ consists of *M*_1_ and his submembrane to hold the output;

*ρ* = 0, 1, 2;

$R=R_{1}\cup R_{M_{1}}$ ;

*R*_1_ = {*r*_1_: (*T* → (*a*, *M*_1_), 2), *r*_2_: (0 → (*b*, *M*_1_), 2), *r*_3_: (1 → (*c*, *M*_1_), 2),

*r*_4_: (*Tn* → *un*(*a*, *M*_1_), 1), *r*_5_: (0*n* → *un*(*b*, *M*_1_), 1), *r*_6_: (1*n* → *un*(*c*, *M*_1_), 1),

*r*_7_: (*sY* → *En*, 1), *r*_8_: (*E* → (*E*, *M*_1_), 1), *r*_9_: (*u* → (*u*, *M*_1_)|_*T*_, 0),

*r*_10_: (*u* → (*u*, *M*_1_)|_0_, 0), *r*_11_: (*u* → (*u*, *M*_1_)|_1_, 0)}

$R_{M_{1}}=\{r_{1}:(af\to A[f],1),r_{2}:(bf\to B[f],1),r_{3}:(cf\to C[f],1),$

*r*_4_: (*a* → (*a*, *in*), 2), *r*_5_: (*b* → (*b*, *in*), 2), *r*_6_: (*c* → (*c*, *in*), 2), *r*_7_: (*aE* → *x*(*E*, *in*), 1),

*r*_8_: (*bE* → *y*(*E*, *in*), 1), *r*_9_: (*cE* → *z*(*E*, *in*), 1), *r*_10_: (*Ax* → 1(*px*, *in*), 1),

*r*_11_: (*Bx* → 0(*qx*, *in*), 1), *r*_12_: (*Cx* → *T*(*rx*, *in*), 1), *r*_13_: (*Ay* → 0(*py*, *in*), 1),

*r*_14_: (*By* → 0(*qy*, *in*), 1), *r*_15_: (*Cy* → 0(*ry*, *in*), 1), *r*_16_: (*Az* → *T*(*pz*, *in*), 1),

*r*_17_: (*Bz* → 0(*qz*, *in*), 1), *r*_18_: (*Cz* → 1(*rz*, *in*), 1), *r*_19_: (*xf* → [*f*], 1),

*r*_20_: (*yf* → [*f*], 1), *r*_26_: (*ru* → *C*(*u*, *in*), 1), *r*_27_: (0^2^ → 0, 1), *r*_28_: (0*T* → *T*, 1),

*r*_29_: (01 → 1, 1), *r*_30_: (*T*^2^ → 1(*T*, *in*), 1), *r*_31_: (*T*1 → 0, 1), *r*_32_: (1^2^ → *T*(1, *in*), 1)}

In this case, the correspondence of the objects is as follows.

The multiplicand numbers *T*, 0, and 1 are represented as objects *a*, *b*, and *c* when they enter membrane *M*_1_ from membrane 1. They reach the membrane where object *f* is located, and are converted to objects *A*, *B* and *C* after reacting with *f*. They are converted to objects *p*, *q*, and *r* when they are multiplied by the multiplier and move toward the inner membrane.

The multiplier numbers enter the membrane *M*_1_ also represented by the objects *a*, *b*, and *c*. When they arrive in the membrane where *E* is located, they are represented by the objects *x*, *y*, and *z* after reacting with *E*. Objects *s*, *f*, *E*, and *Y* act in the same way as addition. Object *n* is to control the generation of *u*, which converts the shifted multiplicands *p*, *q*, and *r* into the objects *A*, *B*, and *C*.

Let us assume that the multiplicand is *a*_*n*_*a*_*n*−1_…*a*_1_ and the multiplier is *b*_*m*_*b*_*m*−1_…*b*_1_. We illustrate the use of the rules in Π* by multiplying two numbers.

**(1) Input of the multiplicand**:

We input one bit of the multiplicand from low to high every two time slices. First, object *a*_1_ is sent into membrane 1.

- **Case 1**: *a*_1_ = *T*, executing rule *r*_1_ in *R*_1_, object *T* is converted to *a* and is sent into membrane *M*_1_. Object *a* is consumed with *f* (*R*_*M*_: *r*_1_), *a* is converted to *A* to stay in membrane *M*_1_ and a new membrane *M*_2_ is generated, and *f* enters into membrane *M*_2_.
- **Case 2**: *a*_1_ = 0, executing rule *r*_2_ in *R*_1_, object 0 is converted to *b* and is sent into membrane *M*_1_. Object *b* is consumed with *f* (*R*_*M*_: *r*_2_), *b* is converted to *B* to stay in membrane *M*_1_ and a new membrane *M*_2_ is generated, and *f* enters into membrane *M*_2_.
- **Case 3**: *a*_1_ = 1, executing rule *r*_3_ in *R*_1_, object 1 is converted to *c* and is sent into membrane *M*_1_. Object *c* is consumed with *f* (*R*_*M*_: *r*_3_), *c* is converted to *C* to stay in membrane *M*_1_ and a new membrane *M*_2_ is generated, and *f* enters into membrane *M*_2_.

The rules for the execution of the multiplicand *a*_*i*_ (1 < *i* < *n*) are similar to the rules for *a*_1_. Object *a*_*i*_ is sent into membrane 1 first, then it is converted to object *a*, *b* or *c* into membrane *M*_1_. It continues into the inner membrane until it reaches membrane *M*_*i*_ where it is consumed with *f* to produce object *A*, *B* or *C*. The highest bit of multiplicand *a*_*n*_ is sent in membrane 1 with object *s*, which is used to indicate that the multiplicand has been completely imported. Rule *r*_7_ in *R*_1_ is applied, object *s* is consumed with *Y* to produce *E* and *n*, signaling that it is ready to send in the multiplier. At the same time, *E* is sent into membrane *M*_1_, and *n* stays in membrane 1 for the production of *u*.

**(2) Input of the multiplier**:

Object *b*_1_ is sent into membrane 1.

- **Case 1**: *b*_1_ = *T*, rule *r*_4_ in *R*_1_ is applied, object *T* and *n* are consumed to produce *u*, *n*, and *a*, object *a* is sent into membrane *M*_1_. Rule *r*_7_ in *R*_*M*_ is applied, object *a* is converted to *x* to stay in membrane *M*_1_, and *E* enters membrane *M*_2_.
- **Case 2**: *b*_1_ = 0, rule *r*_5_ in *R*_1_ is applied, object 0 and *n* are consumed to produce *u*, *n*, and *b*, object *b* is sent into membrane *M*_1_. Rule *r*_8_ in *R*_*M*_ is applied, object *b* is converted to *y* to stay in membrane *M*_1_, and *E* enters membrane *M*_2_.
- **Case 3**: *b*_1_ = 1, rule *r*_6_ in *R*_1_ is applied, object 1 and *n* are consumed to produce *u*, *n*, and *c*, object *c* is sent into membrane *M*_1_. Rule *r*_9_ in *R*_*M*_ is applied, object *c* is converted to *z* to stay in membrane *M*_1_, and *E* is sent into membrane *M*_2_.

The multiplier *b*_*i*_ (2 ≤ *i* ≤ *m*) performs similar rules to *b*_1_. Object *b*_*i*_ is sent into membrane 1, then it is converted to object *a*, *b*, or *c* into membrane *M*_1_. It continues into the inner membrane until it reaches membrane *M*_*i*_ where it is consumed with *E* and produces object *x*, *y*, or *z*.

**(3) Multiplication**:

Multiplication of two numbers involves both multiplication and addition operations, and these rules are performed simultaneously. When object *b*_1_ is sent into membrane *M*_1_, it is prepared for multiplication operations with *a*_*n*_*a*_*n*−1_…*a*_1_.

Multiplication possibilities of *b*_1_ × *a*_1_ are as follows:

- **Case 1**: *T* × *T* = 1 (*R*_*M*_: *r*_10_);
- **Case 2**: *T* × 0 = 0 (*R*_*M*_: *r*_13_);
- **Case 3**: *T* × 1 = *T* (*R*_*M*_: *r*_16_);
- **Case 4**: 0 × *T* = 0 (*R*_*M*_: *r*_11_);
- **Case 5**: 0 × 0 = 0 (*R*_*M*_: *r*_14_);
- **Case 6**: 0 × 1 = 0 (*R*_*M*_: *r*_17_);
- **Case 7**: 1 × *T* = *T* (*R*_*M*_: *r*_12_);
- **Case 8**: 1 × 0 = 0 (*R*_*M*_: *r*_15_);
- **Case 9**: 1 × 1 = 1 (*R*_*M*_: *r*_18_).

Taking the case of “*T* × *T* = 1” as an example, Rule *R*_*M*_: *r*_10_ signifies that object *A* is consumed with *x* to produce 1, which is retained in the original membrane. Object *A* is converted to *p* and sent into membrane *M*_2_ along with object *x*. The conversion of object *A* to *p* and its movement into the inner membrane serves to: 1) Avoid confusion with the multiplicand object in the next layer of the membrane; 2) Allow the result of the multiplication to directly contribute to addition operations in the original membrane. After the input of *b*_2_, object *u* will first be sent to membrane *M*_1_ (*R*_*M*_: *r*_9_ ∼ *r*_11_), where *u* will convert *p* back to *A* (*R*_*M*_: *r*_24_) before *b*_2_ is sent into membrane *M*_2_, enabling *b*_2_ to multiply with object *A*.

Object *b*_1_ is sent to membrane *M*_2_ to multiply by *a*_2_. The result of the calculation is retained in the original membrane, *a*_2_ is converted and sent into membrane *M*_3_ along with the multiplicand *b*_1_. This process continues until *b*_1_ enters membrane *M*_*n*_. After multiplication by *a*_*n*_, the converted *b*_1_ and *a*_*n*_ arrive at membrane *M*_*n*+1_, where *f* is located. Object *b*_1_ is consumed with *f* (*R*_*M*_: *r*_19_ ∼ *r*_21_), producing a new membrane *M*_*n*+2_, and *f* is sent into the new membrane to prevent spillage.

After the input of *b*_2_, object *u* will be sent into membrane *M*_1_ first (*R*_*M*_: *r*_9_ ∼ *r*_11_). Object *u* will convert the multiplicand from object *p*, *q*, or *r* to object *A*, *B*, or *C* (*R*_*M*_: *r*_24_ ∼ *r*_26_) before *b*_2_ is sent into membrane *M*_2_. In membrane *M*_2_, object *b*_2_ multiplies with *a*_1_, and the product is then added to the result of *b*_1_ × *a*_1_ according to rules (*R*_*M*_: *r*_27_ ∼ *r*_32_). *b*_2_ then continues into the inner membrane for further reactions. The next objects *b*_*i*_ (3 ≤ *i* ≤ *m*) also react according to the earlier described rules.

The final result is stored sequentially in membranes *M*_1_, …, *M*_*k*_ (*k* ≥ 1). The multiplication example in Section 4.1.2 demonstrates the concrete implementation of the rules. In the multiplication P System Π*, symmetric ternary numbers “*n* × *m*” require at most 3*n* + 4*m* + 3 time slices to complete the multiplication operation.

### 3.3 Division

According to the definition of cell-like P Systems, a division arithmetic P System based on symmetrical ternary can be defined as:

$$\begin{array}{c} \Pi^{/}=\left(V,\mu ,\omega_{1},\omega_{M_{1}},R,\rho ,i_{0}\right) \end{array}$$ (6)

Where:

*V* = {*a*, *b*, *c*, *T*, 0, 1, *A*, *B*, *C*, *K*, *M*, *N*, *H*, *V*, *k*, *x*, *i*, *v*, *r*, *u*, *g*, *s*, *f*, *e*, *E*, *Y*, *X*};

$\mu ={[}_{1}{[}_{M_{1}}{]}_{M_{1}}{]}_{1}$ ;

*ω*_1_ = {*Y*};

$\omega_{M_{1}}=\{f\}$ ;

*i*_0_ consists of *M*_1_ and his submembrane to hold the output;

*ρ* = 0, 1, 2, 3, 4, 5;

$R=R_{1}\cup R_{M_{1}}$ ;

*R*_1_ = {*r*_1_: (*T* → (*a*, *M*_1_)|_*Y*_, 1), *r*_2_: (0 → (*b*, *M*_1_)|_*Y*_, 1), *r*_3_: (1 → (*c*, *M*_1_)|_*Y*_, 1),

*r*_4_: (*T* → (*c*, *M*_1_), 2), *r*_5_: (0 → (*b*, *M*_1_), 2), *r*_6_: (1 → (*a*, *M*_1_), 2),

*r*_7_: (*sY* → *E*(*s*, *M*_1_), 1), *r*_8_: (*E* → (*E*, *M*_1_), 1), *r*_9_: (*sK* → *eK*, 2),

*r*_10_: (*K* → *Nk*|_*e*_, 1), *r*_11_: (*ke* → *e*(*k*, *M*_1_), 1), *r*_12_: (*N* → *M*|_*e*_, 1),

*r*_13_: (*M* → *H*|_*e*_, 1), *r*_14_: (*H* → *K*|_*e*_, 1), *r*_15_: (*xe* → *X*(*V*, *in*), 0),

*r*_16_: (*vX* → *XV*(*k*, *in*), 1), *r*_17_: (*V* → (*V*, *in*), 1), *r*_18_: (*kX* → *X*, 0)}

$R_{M_{1}}=\{r_{1}:(af\to T[f],1),r_{2}:(bf\to 0[f],1),r_{3}:(cf\to 1[f],1),$

*r*_4_: (*a* → (*a*, *in*), 2), *r*_5_: (*b* → (*b*, *in*), 2), *r*_6_: (*c* → (*c*, *in*), 2),

*r*_7_: (*aE* → *A*(*E*, *in*), 1), *r*_8_: (*bE* → *B*(*E*, *in*), 1), *r*_9_: (*cE* → *C*(*E*, *in*), 1),

*r*_10_: (0*k* → *T*, (*k*, *in*)|_*A*_, 3), *r*_11_: (0*k* → 0, (*k*, *in*)|_*B*_, 3), *r*_12_: (0*k* → 1, (*k*, *in*)|_*C*_, 3),

*r*_13_: (*Tk* → 1, (*Tk*, *in*)|_*A*_, 3), *r*_14_: (*Tk* → *T*, (*k*, *in*)|_*B*_, 3), *r*_15_: (*Tk* → 0, (*k*, *in*)|_*C*_, 3),

*r*_16_: (1*k* → 0, (*k*, *in*)|_*A*_, 3), *r*_17_: (1*k* → 1, (*k*, *in*)|_*B*_, 3), *r*_18_: (1*k* → *T*, (1*k*, *in*)|_*C*_, 3),

*r*_19_: (0^2^ → 0, 2), *r*_20_: (0*T* → *T*, 2), *r*_21_: (01 → 1, 2), *r*_22_: (*T*^2^ → 1(*T*, *in*), 2),

*r*_23_: (*T*1 → 0, 2), *r*_24_: (1^2^ → *T*(1, *in*), 2), *r*_25_: (*s* → (*s*, *in*), 1),

*r*_26_: (*s* → *g*(*u*, *out*)|_*f*_, 0), *r*_27_: (0*k* → 0(*k*, *in*), 4), *r*_28_: (*Tk* → *T*(*k*, *in*), 4),

*r*_29_: (1*k* → 1(*k*, *in*), 4), *r*_30_: (*u*0 → *δu*, 1), *r*_31_: (*u*0*k* → *δu*(*k*, *in*), 0),

*r*_32_: (*u* → *x*|_*A*_, 0), *r*_33_: (*u* → *x*|_*B*_, 0), *r*_34_: (*u* → *x*|_*C*_, 0), *r*_35_: (*xk* → (*ix*, *out*), 1),

*r*_36_: (*x* → (*x*, *out*), 2), *r*_37_: (0*i* → 1(*i*, *out*)|_*A*_, 1), *r*_38_: (0*i* → 0(*i*, *out*)|_*B*_, 1),

*r*_39_: (0*i* → *T*(*i*, *out*)|_*C*_, 1), *r*_40_: (*Ti* → 0(*i*, *out*)|_*A*_, 1), *r*_41_: (*Ti* → *T*(*i*, *out*)|_*B*_, 1),

*r*_42_: (*Ti* → (*T*, *in*)1(*i*, *out*)|_*C*_, 1), *r*_43_: (1*i* → (1, *in*)*T*(*i*, *out*)|_*A*_, 1),

*r*_44_: (1*i* → 1(*i*, *out*)|_*B*_, 1), *r*_45_: (1*i* → 0(*i*, *out*)|_*C*_, 1),

*r*_46_: (*v* → (*v*, *out*)|_1*A*_, 1), *r*_47_: (*v* → (*v*, *out*)|_0*B*_, 1), *r*_48_: (*v* → (*v*, *out*)|_*TC*_, 1),

*r*_49_: (*v* → (*v*, *out*)|_1*B*_, 1), *r*_50_: (*v* → (*v*, *out*)|_0*C*_, 1), *r*_51_: (*v* → (*r*, *out*)|_1*C*_, 1),

*r*_52_: (*v* → (*r*, *out*)|_0*A*_, 1), *r*_53_: (*v* → (*r*, *out*)|_*TB*_, 1), *r*_54_: (*v* → (*r*, *out*)|_*TA*_, 1),

*r*_55_: (*r* → (*r*, *out*), 1), *r*_56_: (*V* → (*v*, *out*)|_*f*_, 0), *r*_57_: (*V* → (*V*, *in*), 1),

*r*_58_: (*gk* → [1*g*], 0), *r*_59_: (1*g* → 1[*g*], 1), *r*_60_: (*fk* → *f*(1, *in*), 1)}

In this case, the correspondence of some substances is as follows. The dividends *T*, 0, 1 are represented as the objects *a*, *b*, *c* when they enter the membrane *M*_1_ from the membrane 1. When they arrive in the membrane where *f* is located, and are represented as the objects *T*, 0, 1 after reacting with *f*. The divisors *T*, 0, 1 enter membrane *M*_1_ as objects *c*, *b*, *a*, and arrive in the membrane where *E* is located. They are represented as objects *A*, *B*, *C* after reacting with *E*. Objects *s*, *f*, *E*, and *Y* function in the same way as addition. Objects *K*, *M*, *N*, *H*, *e* are used to control the generation of object *k*. Object *u* is used to determine whether the digits of the dividend are equal to the divisor.

Object *u* is converted to *x* when it encounters the divisor. Object *k* produced after the dividend and the divisor digits are the same, will be consumed with *x* and *i* produced. Object *i* converts the dividend to the state it was in when it was just the same number of digits as the divisor. Send *V* from membrane 1 into the innermost submembrane of membrane *M*_1_. If the dividend is greater than the divisor, return *v* to membrane 1 to produce *k*, and continue with the addition; if it is less than that, return *r* to indicate that no more addition can be performed. At the end of the reaction, the quantity of *k* is the decimal representation of the quotient, and *g* converts the quantity of *k* to symmetric ternary.

In this paper, division of two numbers is actually accomplished by iterative subtraction, where the divisor is subtracted from the dividend. The cycle of subtraction continues until the dividend is less than the divisor. The number of rounds of subtraction is the quotient, and the remaining dividend that cannot be subtracted anymore is the remainder. In symmetric ternary, subtraction is converted to addition by simply changing the non-zero bit of the divisor to its opposite, i.e., 1 to *T* and *T* to 1. So, we can change iterative subtraction to iterative addition. The following modules are used in the Division P System:

- **Module 1** (*R*_1_: *r*_1_ ∼ *r*_8_ and *R*_*M*_: *r*_1_ ∼ *r*_9_) is the input of dividend and divisor. The dividend is input and stored in each layer of the membrane as “T, 0, 1”, and the non-zero bit of the divisor is turned into its opposite and stored in each layer of the membrane as “A, B, C”.
- **Module 2** (*R*_*M*_: *r*_10_ ∼ *r*_24_) is the iterative addition of the dividend and the opposite of the divisor. When the number of bits of the dividend is greater than the number of bits of the divisor, no judgment is required to perform the addition operation.
- **Module 3** (*R*_*M*_: *r*_37_ ∼ *r*_45_) is a module that restores the dividend to the state when the number of digits of the dividend is the same as the number of digits of the divisor. And at the same time, object *k* is consumed in its entirety, and the generation of *k* is suppressed in Membrane 1.
- **Module 4** (*R*_*M*_: *r*_46_ ∼ *r*_57_) is a module that determines whether the dividend is greater than the divisor. If the dividend is greater than the divisor, the addition continues; if it is less, the reaction stops.

Let us assume that the dividend is *a*_*n*_*a*_*n*−1_…*a*_1_ and the divisor is *b*_*m*_*b*_*m*−1_…*b*_1_ (where *n* ≥ *m*), and we illustrate the use of the rules in Π^/^ below by dividing two numbers.

**(1) Input of the dividend**:

First, *a*_1_ is sent into membrane 1.

- **Case 1**: *a*_1_ = *T*, rule *r*_1_ in *R*_1_ is applied, and object *T* is converted to *a* in the presence of *Y* and is sent into membrane *M*_1_. Rule *r*_1_ in *R*_*M*_ is executed, and *a* is converted into *T* while a new membrane *M*_2_ is created, and *f* enters membrane *M*_2_.
- **Case 2**: *a*_1_ = 0, rule *r*_2_ in *R*_1_ is applied, and object 0 is converted to *b* in the presence of *Y* and is sent into membrane *M*_1_. Rule *r*_2_ in *R*_*M*_ is executed, and *b* is converted into 0 while a new membrane *M*_2_ is created, and *f* enters membrane *M*_2_.
- **Case 3**: *a*_1_ = 1, rule *r*_3_ in *R*_1_ is applied, and object 1 is converted to *c* in the presence of *Y* and is sent into membrane *M*_1_. Rule *r*_3_ in *R*_*M*_ is executed, and *c* is converted into 1 while a new membrane *M*_2_ is created, and *f* enters membrane *M*_2_.

Object *a*_*i*_ (1 < *i* < *n*) is sent into membrane 1 and converted to object *a*, *b*, or *c* into membrane *M*_1_. Then it continues into the inner membrane until it reaches membrane *M*_*i*_ where it is consumed with *f* and is converted to object *T*, 0 or 1. The last bit of the dividend *a*_*n*_ is sent in with object *s* to indicate that the dividend has been fully entered. When object *s* enters membrane 1, rule *r*_7_ in *R*_1_ is applied. Object *E* is produced, signaling the system that it is ready to enter the divisor, while *s* is sent into membrane *M*_1_. After that, object *s* continues into the inner membrane (*R*_*M*_: *r*_25_) until it reaches membrane *M*_*n*_. In membrane *M*_*n*_, object *s* is converted to *u* and *g* (*R*_*M*_: *r*_26_) catalyzed by *f*. Object *u* is exported to membrane *M*_*n*−1_ (*R*_*M*_: *r*_26_), and *g* stays in membrane *M*_*n*_.

**(2) Input of the divisor**:

Object *b*_1_ is sent into membrane 1:

- **Case 1**: *b*_1_ = *T*. Rule *r*_4_ in *R*_1_ is executed, and object *T* is converted to *c* in membrane *M*_1_. Then, rule *r*_9_ in *R*_*M*_ is applied, object *c* is converted to *C*, and *E* enters membrane *M*_2_.
- **Case 2**: *b*_1_ = 0. Rule *r*_5_ in *R*_1_ is executed, and object 0 is converted to *b* in membrane *M*_1_. Then, rule *r*_8_ in *R*_*M*_ is applied, object *b* is converted to *B*, and *E* enters membrane *M*_2_.
- **Case 3**: *b*_1_ = 1. Rule *r*_6_ in *R*_1_ is executed, and object 1 is converted to *a* in membrane *M*_1_. Then, rule *r*_7_ in *R*_*M*_ is applied, object *a* is converted to *A*, and *E* enters membrane *M*_2_.

Object *b*_*i*_ (2 ≤ *i* ≤ *m*) is sent into membrane 1, then it is converted to object *a*, *b*, or *c* into membrane *M*_1_. It continues into the inner membrane until it reaches membrane *M*_*i*_ where it is consumed with *E* to produce object *A*, *B*, or *C*. The highest bit of the divisor *b*_*m*_ is sent into membrane 1 with object *s*. When *s* is sent into membrane 1, rule *r*_9_ in *R*_1_ is applied, object *s* is consumed with *K* to produce *E* and *K*, which is used to produce object *k*.

**(3) Division**:

When the divisor is fully sent into the system, object *k* is produced in Membrane 1 (*R*_1_: *r*_9_ ∼ *r*_10_), and *k* is sent into membrane *M*_1_ to trigger addition. Then one *k* is produced every three time slices into membrane *M*_1_ (*R*_*M*_: *r*_10_ ∼ *r*_14_).

The possibilities of *a*_1_ + *b*_1_ are as follows:

- **Case 1**: 0 + *T* = *T* (*R*_*M*_: *r*_10_);
- **Case 2**: 0 + 0 = 0 (*R*_*M*_: *r*_11_);
- **Case 3**: 0 + 1 = 1 (*R*_*M*_: *r*_12_);
- **Case 4**: *T* + *T* = *T*1 (*R*_*M*_: *r*_13_);
- **Case 5**: *T* + 0 = 0 (*R*_*M*_: *r*_14_);
- **Case 6**: *T* + 1 = 0 (*R*_*M*_: *r*_15_);
- **Case 7**: 1 + *T* = 0 (*R*_*M*_: *r*_16_);
- **Case 8**: 1 + 0 = 1 (*R*_*M*_: *r*_17_);
- **Case 9**: 1 + 1 = 1*T* (*R*_*M*_: *r*_18_).

For cases 4 and 9 that generate feeds, the high bit of the result is sent into the inner membrane and the low bit remains in the original membrane.

Taking “0+T = T” as an example, the rule *R*_*M*_: *r*_10_ indicates that 0 is consumed with *k* in the catalysis of *A*, generating *T* to remain in membrane *M*_1_ and *k* to enter membrane *M*_2_. The divisor, serving as a catalyst, remains unchanged to ensure that the size of the dividend decreases while the divisor remains the same.

When the first round of addition is completed and the first object *k* reaches the membrane *M*_*n*+1_, where objects *g* and *f* are also present. The rule *r*_58_ in *R*_*M*_ is executed to convert *k* to 1 and create a new membrane *M*_*n*+2_, sending 1 and *g* into membrane *M*_*n*+2_. Then, rule *r*_59_ in *R*_*M*_ creates a new membrane *M*_*n*+3_ and sends *g* into membrane *M*_*n*+3_ to prevent result overflow. Thereafter, object *f* converts the *k* of membrane *M*_*n*+1_ to 1 (*R*_*M*_: *r*_60_), sending it into membrane *M*_*n*+2_ and converting the quotient to a symmetric ternary number. Object *g* encountering object 1 creates a new membrane, preventing overflow.

As addition proceeds, the dividend *a*_*n*_*a*_*n*−1_…*a*_1_ decreases, and when *a*_*n*_ = 0, object *u* dissolves the membrane *M*_*n*_ (*R*_*M*_: *r*_30_). This continues until *u* dissolves the membrane *M*_*m*+1_, and encounters the divisor (*R*_*M*_: *r*_32_ ∼ *r*_34_). At this point, the bits of the dividend and the divisor are the same. Further additions should then check if the dividend is greater than the divisor, allowing addition to continue only if this condition is met. However, membrane 1 still produces one *k* every three time slices, which requires reversing the additions done after the numbers have the same number of digits and eliminating the *k* produced, sending a signal to stop *k* production in membrane 1.

When the digits of the dividend and divisor align, object *u* is converted to *x*. *x* is then transported out of the membrane (*R*_*M*_: *r*_36_) until it encounters *k* or reaches membrane 1. If *x* meets *k*, rule *R*_*M*_: *r*_35_ is enacted to produce *i* and *x*. Object *i* reverses the addition performed for this *k* (*R*_*M*_: *r*_37_ ∼ *r*_45_), and *x* continues to membrane 1. Upon reaching, rule *R*_1_: *r*_15_ is executed, generating *X* and *V*. *X* regulates *k* production, while *V* moves to membrane *M*_*m*+1_, converting to *v* catalyzed by *f* (*R*_*M*_: *r*_56_). *v* assesses whether the dividend exceeds the divisor, returning to membrane 1 if greater, or sending *r* to indicate that no further addition can be done (*R*_*M*_: *r*_46_ ∼ *r*_54_).

When *v* returns to membrane 1, rule *r*_16_ in *R*_1_ is executed, producing *k* and sending it to membrane *M*_1_ for further addition, while *V* continues into membrane *M*_*m*+1_, converted to *v* by *f*. The process iterates until *r* returns to membrane 1, signaling the cessation of reactions.

The remainder of the division is stored in membranes *M*_1_, …, *M*_*i*_, and the quotient in membranes *M*_*i*+1_, …, *M*_*f*_ (*f* > *i* + 1). The division example in Section 4.1.3 allows a more concrete demonstration of the implementation of the rule. In the division P System Π^/^, the symmetric ternary numbers of *n*/*m* (*n* ≥ *m*) bits require the following time slices to implement the division operation: The time slices required by the input module is 3*n* + 3*m* − 2, The time slices required by the iterative addition module is 4*i*, The time slices required by the revert module is 2*m*, The time slices required by the evaluation module is (2*m* + 3)*j*, where *i* + *j* = quotient, *i* is the quotient that results when the number of dividend digits is greater than the divisor, and *j* is the quotient that results when the number of dividend digits is equal to the divisor. The efficiency of the system will be greatly improved when the number of dividend digits is much larger than the divisor.

### 3.4 Comparison of computational efficiency

In this section, we analyse and compare P systems proposed in recent years for basic arithmetic operations, the results are shown in Table 5. The statistics include the number of rule types used for the four basic operations (addition, subtraction, multiplication, and division), and the number of time slices required to complete the operations. In [13, 14], *m* and *n* respectively represent the two decimal numbers used in the operation, and *n* = max {*m*, *n*}. In this paper, m and n respectively represent the two symmetric ternary numbers used in the operation, and *n* = max {*m*, *n*}.

**Table 5:** Time slices required and number of rule types used for four arithmetic approaches.

Article	Rule type	Add	Sub	Mul	Div
[13]	5/5/6/11	O(n)	O(n)	O(m)	O(n)
[14]	2/1/11/12	O(1)	O(1)	O(max (n,m))	linear
[15]	39/39/29/34	linear	linear	linear	linear
[16]	3 or 4/4/11/10	-	-	-	-
This Work	21/21/43/78	O(4m)	O(4m)	O(4m)	O(3n)

Note: The ‘Rule type’ column is represented in the form of A/B/C/D, where A represents the number of rules used for addition. Similarly, B, C, and D denote the number of rule types used for subtraction, multiplication, and division, respectively.

In Table 5, the arithmetic P systems designed in [13–16] are all decimal based. And instead of considering the input of data, the numbers to be computed are directly put into the membranes. In other words, they did not take into account the input of computational data. [13] designed an arithmetic P system based on a multi-layer membrane, and [14] designed an arithmetic P system based on a single membrane In [15] the time slices of the operations is not given, only it is mentioned that the complexity of these operations in P system is “linear”. Since [16] discusses arithmetic operation and arithmetic expression evaluation in transition P system based on rules with priority, no arithmetic operations are performed. Therefore, the time slices required for arithmetic operations is marked with ‘-’.

From the above analysis, it can be seen that this paper not only considers the input of the data, the dynamic generation and dissolution of the membrane, but also introduces the symmetric ternary number system, which eliminates the influence of the operational sign on the calculation. The arithmetic operation P system designed in this paper is more novel and has a wider application scenario.

## 4 Simulation and validation of rules

This section is based on Section 3 to further validate the correctness of the rules in the P System. The rules in Section 3 are simulated and experimented with UPSimulator [27]. The main discussion is the simulation experiments of (1) addition: 1*T*00 + 1*T*1 = 10*T*1; (2) multiplication: 1*TT* × 1*T* = 101; and (3) division: 101/1*T* = 1*TT*.

### 4.1 Examples of symmetric ternary arithmetic operations

#### 4.1.1 Example of addition

Here is an example of “1*T*00 + 1*T*1” to illustrate the implementation of the addition rules.

**Fig 2:**
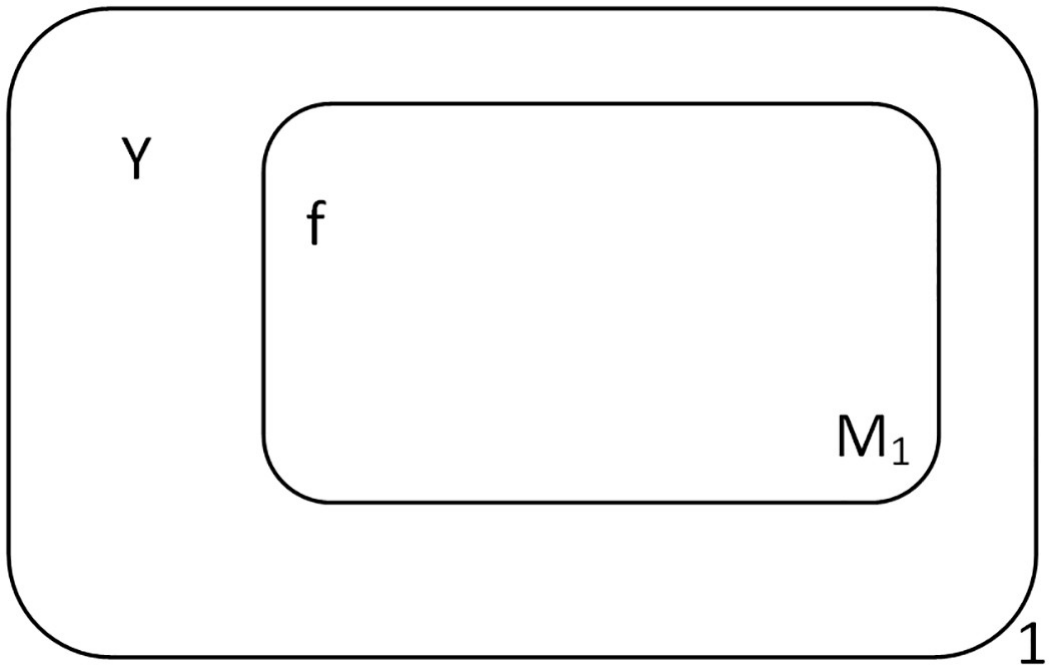
Initial configuration of Π^+^.

The initial state of Π^+^ is shown in Fig 2. Firstly, objects 0, 0, *T*, 1 are sequentially sent into membrane 1 every two time slices. When object 0 is input to membrane 1, it is converted to object *b* and is sent into membrane *M*_1_ immediately. Object *b* is consumed with *f* in membrane *M*_1_ and object 0 produced, at the same time, a new membrane *M*_2_ is created with *f* entering membrane *M*_2_. The next input of object 0 is converted to object *b* and is sent into membrane *M*_2_, which then is consumed with *f* and object 0 produced, and creates a new membrane *M*_3_, with *f* entering membrane *M*_3_. Object *T* is input to membrane 1, it is converted to object *a* and is sent into membrane *M*_3_. Object *a* is consumed with *f* and object *T* produced, and creating a new membrane *M*_4_, with *f* entering membrane *M*_4_.

**Fig 3:**
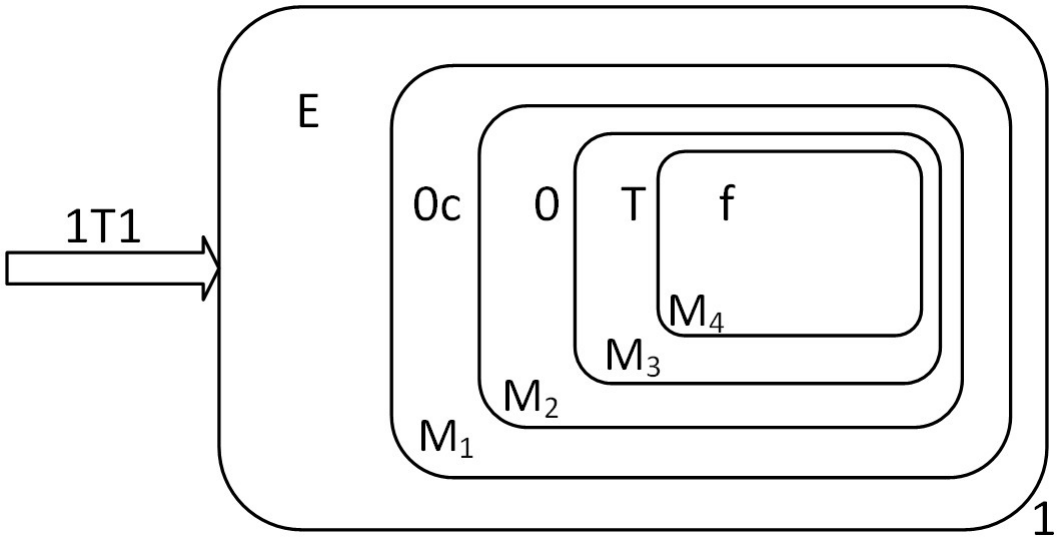
The configuration of addend waiting for input.

When the highest bit object 1 is input, object *s* is input at the same time to indicate the end of the augend number. Objects 1 and *s* are input to membrane 1, and object 1 is converted to object *c* which is sent into membrane *M*_4_. Object *c* is consumed with *f* and object 1 produced, and creates a new membrane *M*_5_, with *f* entering into membrane *M*_5_. Object *s* is consumed with *Y* in membrane 1 and *E* produced, which indicates that the addend number can be input now, and at the same time, object *E* enters into membrane *M*_1_. Fig 3 shows the membrane structure where the augend is input completely and the addend waits to be input.

The lowest bit of the addend, object 1, is sent into membrane 1. Object 1 is converted to *c* and is sent into membrane *M*_1_. Object *c* and *E* are consumed with object 1 produced, and object *E* is sent into membrane *M*_2_. Object 1 is added to the 0 in membrane *M*_1_, and the generated results is 1, which is stored in membrane *M*_1_.

Input the second bit of the addend, object *T*, to membrane 1. Object *T* is converted to *a*, which is sent into membrane *M*_2_, where it is consumed with *E* to generate object *T*. Object *T* and the object 0 in membrane *M*_2_ are consumed with object *T* produced, which is stored in membrane *M*_2_.

Send the last bit of the addend, object 1, to membrane 1. Object 1 is converted to *c*, which is sent into membrane *M*_3_ and reacts with *E* to generate object 1. Object 1 is consumed with object *T* in membrane *M*_3_ and object 0 produced, which is stored in membrane *M*_3_.

**Fig 4:**
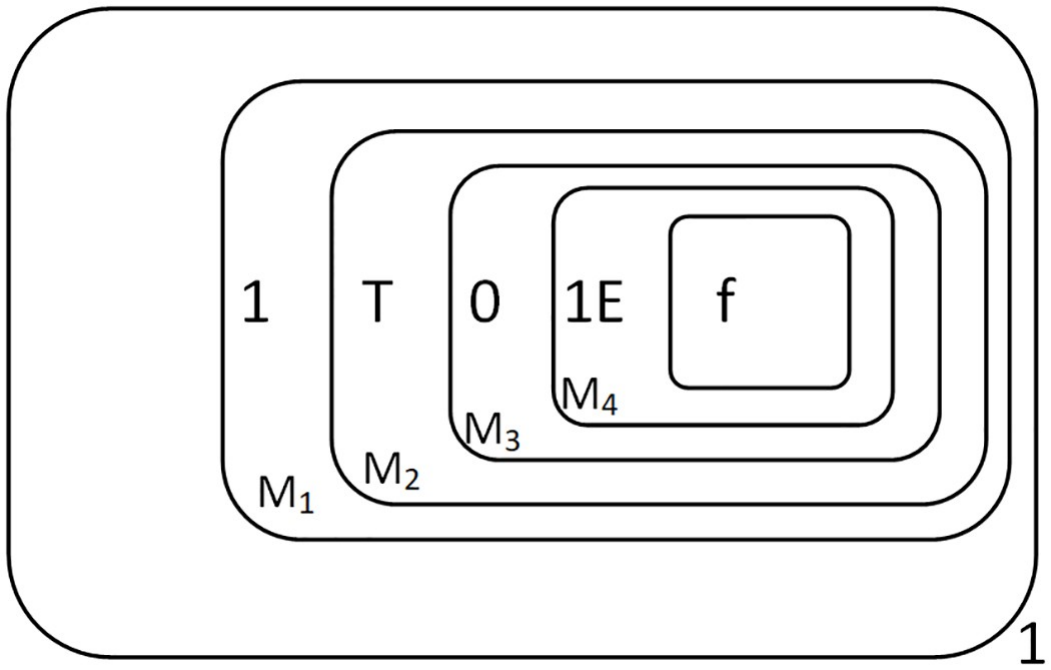
The configuration at completion of addition.

At this point, there are no more rules in the system that can be executed, the system stops, and the obtained result “10T1” is saved in low to high order in *M*_1,…,4_ as shown in Fig 4. The rules of the entire system run in parallel. While the augend is still moving towards the inner membrane and building new membranes, the addend has already been input to start the addition operation. In this way, the whole system can perform the operation quickly.

Tables 6 and 7 show the process of object changes in each membrane during the whole system run. The number of digits of the augend and the addend also has an effect on the time slices. For example, in Table 6, it takes 23 time slices for the augend to be 1*T*00 (four-digit number) and the addend to be 1*T*1 (three-digit number). While in Table 7, it takes 24 time slices for the augend to be 1*T*1 (three-digit number) and the addend to be 1*T*00 (four-digit number). Therefore, when using this arithmetic system, the one with more digits can be selected as the augend to reduce the time slices.

**Table 6:** Process of object changes in each membrane during the addition. (1T00+1T1).

Time Slice	Membrane 1	M1	M2	M3	M4	M5
0	Y	f				
1	0Y	f				
2	Y	bf				
3	Y	0	f			
4	0Y	0	f			
5	T	0b	f			
6	Y	0	bf			
7	TY	0	0	f		
8	Y	0a	0	f		
9	Y	0	0a	f		
10	1sY	0	0	af		
11	E	0c	0	T	f	
12	1	0E	0C	T	f	
13		0cE	0	cT	f	
14		01	0E	T	1	f
15	T	1a	0E	T	1	f
16		1	0E	T	1	f
17		1	0Ea	T	1	f
18	1	1c	0T	TE	1	f
19		1	T	TE	1	f
20		1	cT	TE	1	f
21		1	T	TEc	1	f
22		1	T	T1	1E	f
23		1	T	0	1E	f

**Table 7:** Process of object changes in each membrane during the addition. (1T1+1T00).

Time Slice	Membrane 1	M1	M2	M3	M4	M5	M6
0	Y	f					
1	1Y	f					
2	Y	cf					
3	Y	1	f				
4	TY	1	f				
5	Y	1a	f				
6	Y	1	af				
7	1sY	1	T	f			
8	E	1c	T	f			
9	0	1E	Tc	f			
10		1Eb	T	cf			
11		10	TE	1	f		
12	0	1	TE	1	f		
13		1b	TE	1	f		
14		1	TEb	1	f		
15	T	1	T0	1E	f		
16		1a	T	1E	f		
17		1	Ta	1E	f		
18	1	1	T	1Ea	f		
19		1c	T	1T	Ef		
20		1	cT	0	E	f	
21		1	T	0c	E	f	
22		1	T	0	Ec	f	
23		1	T	0	1	Ef	
24		1	T	0	1	E	f

#### 4.1.2 Example of multiplication

Here is an example of “1*TT* × 1*T*” to illustrate the implementation of the multiplication rules.

**Fig 5:**
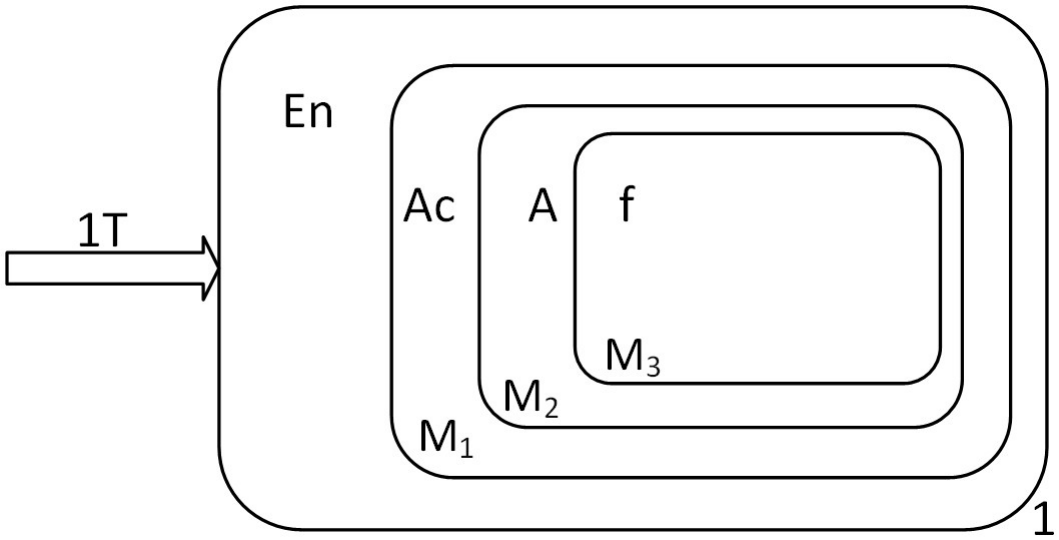
The configuration of Π* when the multiplier is about to be input.

The input of the multiplicand is the same as the augend, so we won’t go into too much detail here. The multiplicand “1*TT*” is finally stored in the form of “*AAC*” in the membranes *M*_1_, *M*_2_, *M*_3_. Object *s* is consumed with Object *Y* in membrane 1 and object *E* produced, at which point the multiplier object *T* can be input, and *E* then enters the membrane *M*_1_. The state of the membrane system when the multiplier is about to be input is shown in Fig 5.

The lowest bit of the multiplier, object *T*, is input. Object *T* is consumed with *n* to generate *u*, and *T* is converted to object *a* into membrane 1. Object *a* is consumed with *E* to produce *x* while *E* enters the membrane *M*_2_. Object *x* is consumed with *A* to produce object 1 which is retained in membrane *M*_1_, and *A* is converted to object *p* which enters membrane *M*_2_ with *x*. In membrane *M*_2_, object *x* is consumed with *A* to produce object 1 which is retained in membrane *M*_2_. Object *A* is converted to *p* which enters membrane *M*_3_ with *x*. In membrane *M*_3_, object *x* is consumed with *C* to produce *T* to be retained in membrane *M*_3_, and *C* is converted to *r* which is sent into membrane *M*_4_ with *x*. Object *x* and *f* in the membrane *M*_4_ are consumed to create a new membrane *M*_5_, while *f* is sent into membrane *M*_5_ to prevent the result from overflowing.

**Fig 6:**
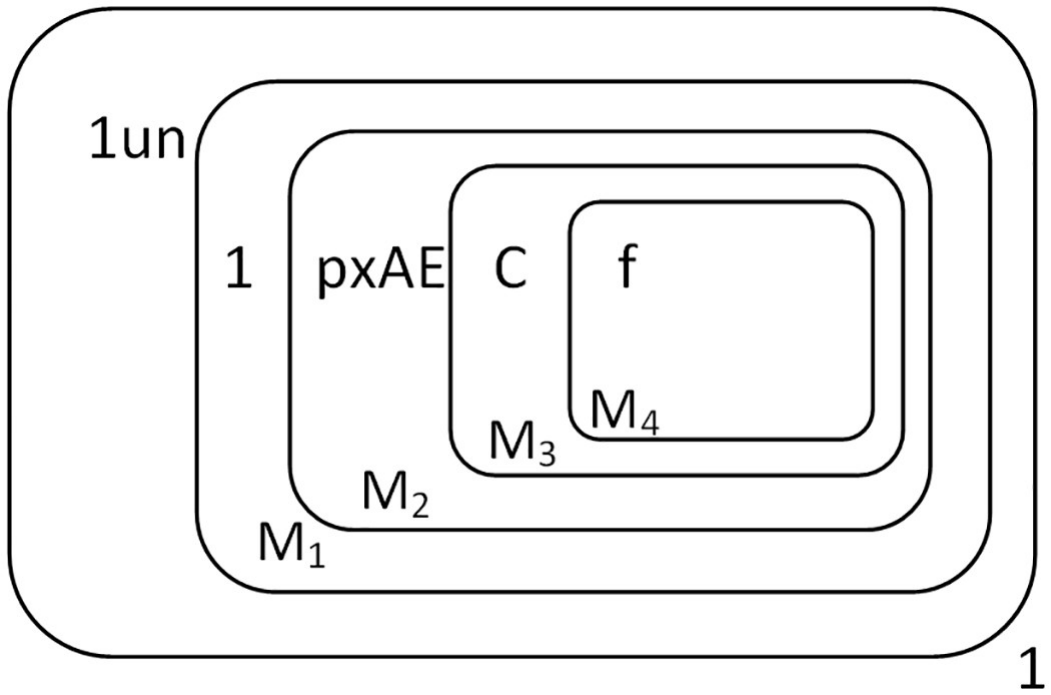
The configuration of Π* when the multiplier is input completely.

At this point, the objects “CCA” are converted to “ppr” and moved one layer into the membrane. In fact, when object *T* is sent into the membrane system to carry out the above biochemical reaction, object 1 is also sent into the membrane to start the calculation. Fig 6 shows the state of the membrane system when object 1 has just been sent into the membrane, and object *T* has just arrived in the membrane *M*_2_ about to undergo the next calculation, and these calculations are in parallel.

When object 1 is input, object *u* will be sent into membrane *M*_1_ and reach membrane *M*_2_, which converts *p* to *A*. It continues to move to the inner membranes, converting *p* and *r* to *A* and *C*. Object 1 is converted to *c* to be sent into membrane *M*_2_, which is consumed with *E* to generate *z*. Object *z* is consumed with *A* to produce *T*, and Object *A* is converted to *p*, which is sent into membrane *M*_3_ with *z*. Object *T* is consumed with the previously generated object 1 and object 0 produced. In membrane *M*_3_, object *z* and *A* are consumed to produce *T*, and then *A* is converted to *p* along with *z* into membrane *M*_4_. Object *T* in membrane *M*_3_ is consumed with the previously generated *T* to produce objects *T*1, with 1 retained in membrane *M*_3_ and *T* as a feed into membrane *M*_4_. In membrane *M*_4_, object *z* is consumed with *C* to produce 1, and then *C* is converted to *r* along with *z* into membrane *M*_5_. Object 1 in membrane *M*_4_ is consumed with the previously fed *T* to generate 0 to be retained in membrane *M*_4_. Object *x* is consumed with *f* to create a new membrane *M*_6_ while *f* is sent into membrane *M*_6_.

**Fig 7:**
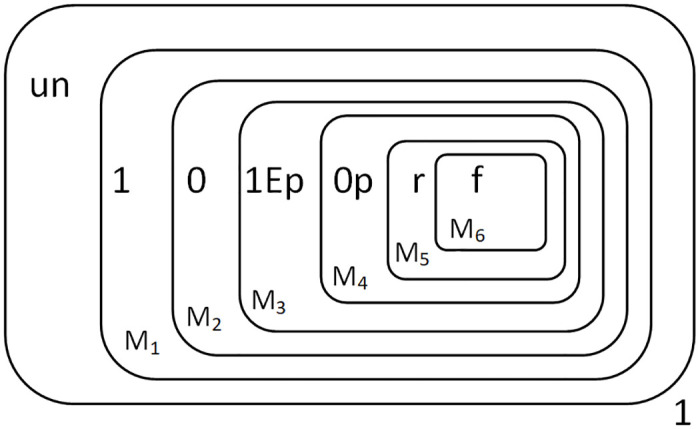
The configuration at the completion of multiplication.

At this point, the reaction was completed. The result “1010” is preserved in the membranes *M*_1_ to *M*_4_ as shown in Fig 7.

Table 8 shows the process of object changes in each membrane as the entire system runs, taking a total of 20 time slices.

**Table 8:** Process of object changes in each membrane during the multiplication.

Time Slice	Membrane 1	M1	M2	M3	M4	M5	M6
0	Y	f					
1	TY	f					
2	Y	af					
3	Y	A	f				
4	TY	A	f				
5	Y	Aa	f				
6	Y	A	af				
7	1sY	A	A	f			
8	En	Ac	A	f			
9	Tn	AE	Ac	f			
10	un	AEa	A	cf			
11	un	Ax	AE	C	f		
12	1un	1	AEpx	C	f		
13	1n	1u	1pE	Cpx	f		
14	un	1c	1pEu	Tp	rxf		
15	un	1	1AEc	Tpu	r	f	
16	un	1	1Az	TAE	ru	f	
17	un	1	1T	TAEpz	C	uf	
18	un	1	0	TTEp	Cpz	f	
19	un	1	0	1Ep	T1p	rzf	
20	un	1	0	1Ep	0p	f	f

#### 4.1.3 Example of division

Here is an example of “101/1*T*” to illustrate the implementation of the division rules.

**Fig 8:**
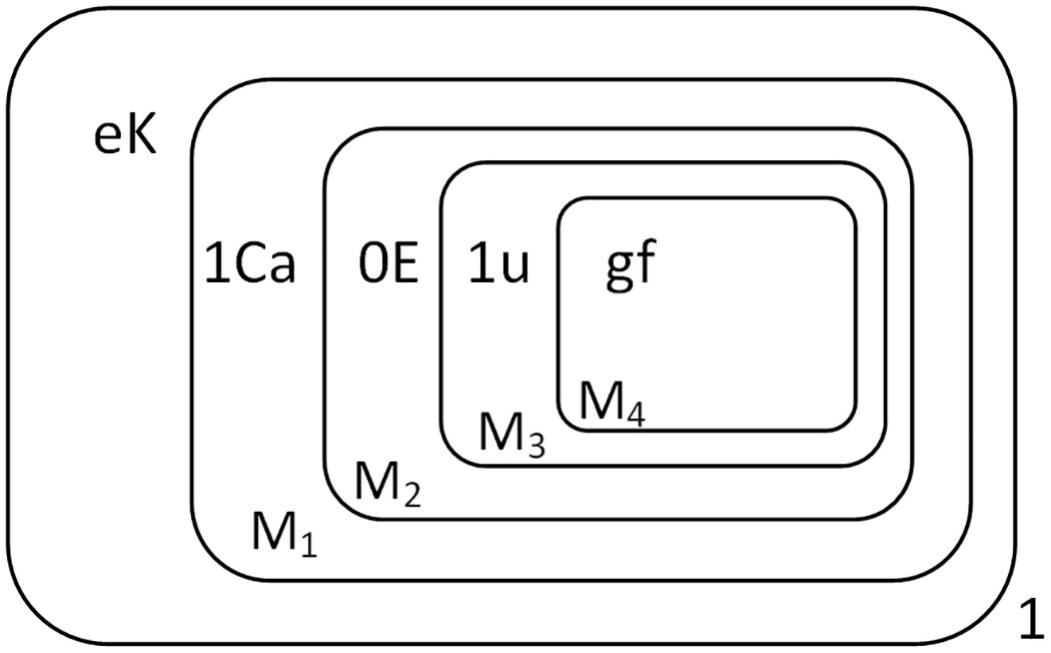
The configuration when all divisors are input to membrane *M*_1_.

The input of the divisor is the same as the augend. When the highest bit of the dividend, object 1, is sent into membrane 1 with object *s*, *s* is consumed with *Y* to produce *E*. At the same time, *s* will enter the membrane *M*_1_ until it reaches the membrane *M*_4_. Object *s* is converted to *u* and *g* catalyzed by *f*. Object *u* is transported to membrane *M*_3_, where the highest bit of the dividend is located. Object *g* remains in membrane *M*_4_. When *E* is detected in the system, the divisor can be entered. First input *T*, Object *T* is converted to *c* into membrane *M*_1_. Object *c* and *E* are consumed to generate *C*, and *E* is sent to membrane *M*_2_. Input 1 and *s*, Object 1 is converted to object *a* into membrane *M*_2_. Object *a* and *E* are consumed to generate *A*, and *E* is sent to membrane *M*_3_. Object *s* and *K* are consumed to generate object *e* and *K*. Object *e* and *K* execute the rules *r*_10_ to *r*_14_ in *R*_1_, and one *k* is produced every four time slices. Then *k* is sent into membrane *M*_1_ to trigger addition operation. The state of the membrane system when all the divisors are sent into membrane *M*_1_ is shown in Fig 8.

The first *k* enters membrane *M*_1_, object 1 and *k* are consumed to generate 1 and *T* in the presence of *C*, then 1 enters membrane *M*_2_ as a feed along with *k*. Object *k* then triggers addition in membrane *M*_2_, and so on, and finally will reach membrane *M*_4_. Object *k* and *g* in membrane *M*_4_ are consumed to create a new membrane *M*_5_, and object 1 and *g* enters membrane *M*_5_. And then 1 and *g* are consumed to create a new membrane *M*_6_ and *g* is sent into membrane *M*_6_.

**Fig 9:**
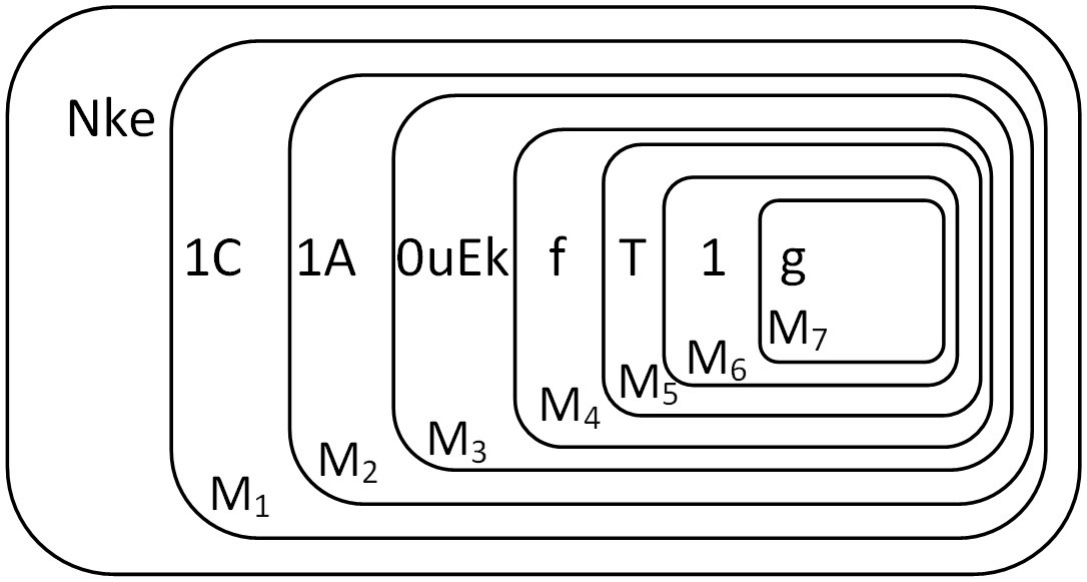
The configuration when the fourth *k* is just generated.

After three rounds of addition, the highest digit of the dividend turns to 0. Object *u* detects 0, dissolves the current membrane, and the objects in membrane *M*_3_ fall into membrane *M*_2_. Object *u* detects the highest digit of the divisor in membrane *M*_2_, indicating that the digits of the dividend and the divisor are now the same. At this point it is not possible to directly perform the addition operation, but to compare the dividend and the divisor before deciding whether it is possible to perform the addition. However, the system continues to produce the object *k*, and the configuration of the system when the fourth *K* is generated is shown in Fig 9.

First, we have to restore the addition operation after the third *k*, and send a signal to membrane 1 to stop producing *k*. Object *u* is converted to *x* catalyzed by *A*, and *k* will be consumed to avoid another addition operation. At the same time, object *i* will be produced, which will restore the dividend to the state where it did three addition operations (*R*_*M*_:*r*_37_ ∼ *r*_45_). Eventually object *i* and *x* will be transported to membrane 1, where *x* is consumed with *e* to prevent the generation of *k*, and produce *X* and *V*. Object *V* is sent into membrane *M*_4_, object *V* is consumed to produce *v* catalyzed by *f*. Object *v* is sent to membrane *M*_3_ to compare the dividend and the divisor. If the dividend is greater than the divisor, then *X* produces *k* to continue the addition.

In this example, the first round of testing determines that the dividend is greater than the divisor, so object *v* is sent to membrane 1. Rule *r*_16_ in membrane 1 is executed, producing *k* which is sent into membrane *M*_1_ to perform addition. At the same time *V* is produced to prepare for the second round of testing. The second round of testing detects that the dividend is still greater than the divisor and sends *v* to membrane 1, performing the same steps as above. The third round of testing judges that no more addition can be performed, returning *r* to membrane 1.

**Fig 10:**
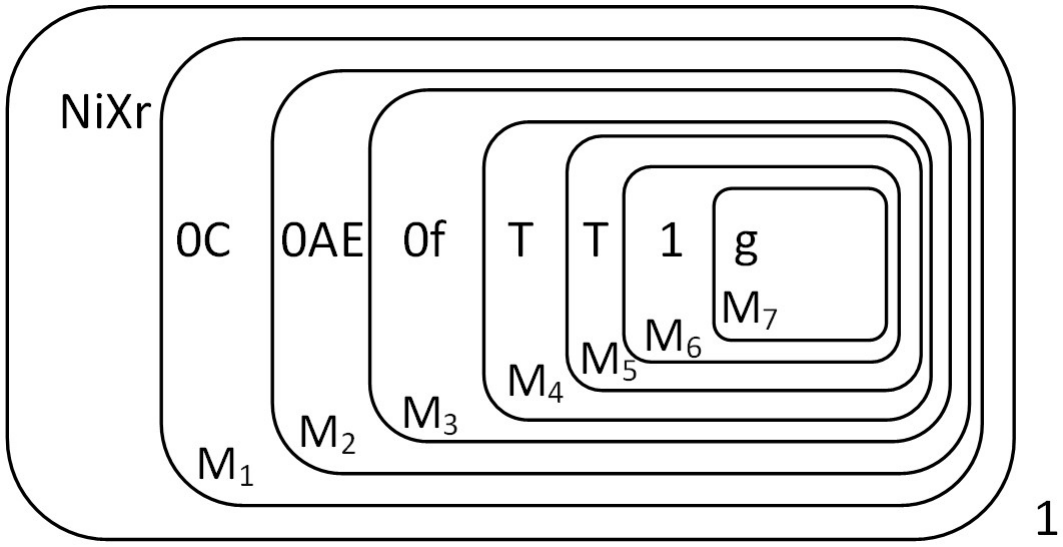
The configuration at the completion of division.

At this point, there are no more rules in the system to execute and the system stops. The result of the quotient is saved in membranes *M*_4,…,6_, and if there is a remainder, it is saved in membranes *M*_1,…,3_. In this example, there is no remainder. The quotient “1TT” is preserved in the membranes *M*_4,…,6_ as shown in Fig 10.

Table 9 shows the process of object changes in each membrane as the division system runs. Due to space constraints, only a portion of the table is shown. It takes a total of 51 time slices.

**Table 9:** Process of object changes in each membrane during the division.

Time Slice	Membrane 1	M1	M2	M3	M4	M5	M6	M7	M8
…	…	…							
12	1sK	1C	0E	1	sf				
13	eK	1Ca	0E	1u	gf				
14	Nke	1C	0Ea	1u	gf				
15	Me	1Ck	0A	1uE	gf				
16	He	TC	0A1k	1uE	gf				
17	Ke	TC	1Ak	1uE	gf				
18	Nke	TC	0A	1uEk	gf				
19	Me	TCk	0A	1uE	gfk				
20	He	0C	0Ak	1uE	f	1g			
21	Ke	0C	TA	1uEk	f	1	g		
…	…	…	…	…	…	…	…		
26	Nke	1C	1A	0uEk	f	T	1	g	
27	Me	1Ck	1AuE		fk	T	1	g	
28	He	TC	1k 1AxE		f	T	1	g	
29	Ke	TCix	TAE		1f	0	1	g	
30	Nke ix	1C	TTAE		1f	0	1	g	
31	NkiX	1CV	1AE		1Tf	0	1	g	
32	NiX	1C	1AEV		0f	0	1	g	
33	NiX	1C	1AE		V0k	0	1	g	
34	NiX	1C	1AEv		0f	0	1	g	
35	NiX	1Cv	1AE		0f	0	1	g	
36	NiXv	1C	1AE		0f	0	1	g	
37	NiXV	1Ck	1AE		0f	0	1	g	
38	NiX	TCV	1k 1AE		0f	0	1	g	
…	…	…	…		…	…	…	…	
51	NiXr	0C	0AE		0f	T	T	1	g

### 4.2 Simulation of addition

For the simulation of Π^+^, the rules in Π^+^ are described in UPLanguage [27]. The membrane class “M” (i.e., membrane 1) is defined to contain the membrane class “Add”, membrane class “B” and membrane class “C”. Membrane classes B and C are used to input the augend and addend respectively. The overall membrane structure is as follows:


Environment {
    Membrane M m1 {
        Object Y;
        Membrane Add A1 {
            Object f;
        }
        Membrane B B1 {
            Object O, B, T, I, s;
        }
        Membrane C C1 {
            Object C, T, I;
        }
    }
}


Where ‘Object’ is used to specify the objects used in the simulation. The above rules indicate that initially the membrane *m*1 contains one object *Y*, the membrane *A*_1_ contains one object *f*, the membrane *B*_1_ contains objects *O*, *B*, *T*, *I*, *s*, and the membrane *C*_1_ contains objects *C*, *T*, *I*. It should be noted that the UPS can’t use numbers to represent the objects, so we use object *I* instead of 1, object *O* instead of 0. When the system starts running, it will output objects *O*, *O*, *T*, and *Is* into membrane *m*_1_ every two time slices from membrane *B*. Object *s* and *Y* are converted to *E* and enters all submembranes. When Object *E* enters membrane *C*, Membrane *C* begins to output addend numbers.

Here is a brief description of the format in which the rules are written in UPS.

- **Rule** *r*_1_: *T* → (*a*, in all), 1; this means that object *T* is converted to object *a* and enters all submembranes with priority 1.
- **Rule** *r*_2_: *af* → *T*Add:*a*{*f*}, 1; this denotes that object *a* and *f* are converted to object *T* and creates a new membrane that inherits the rules of the Add membrane class and that object *f* goes into the newly created membrane.

**Fig 11:**
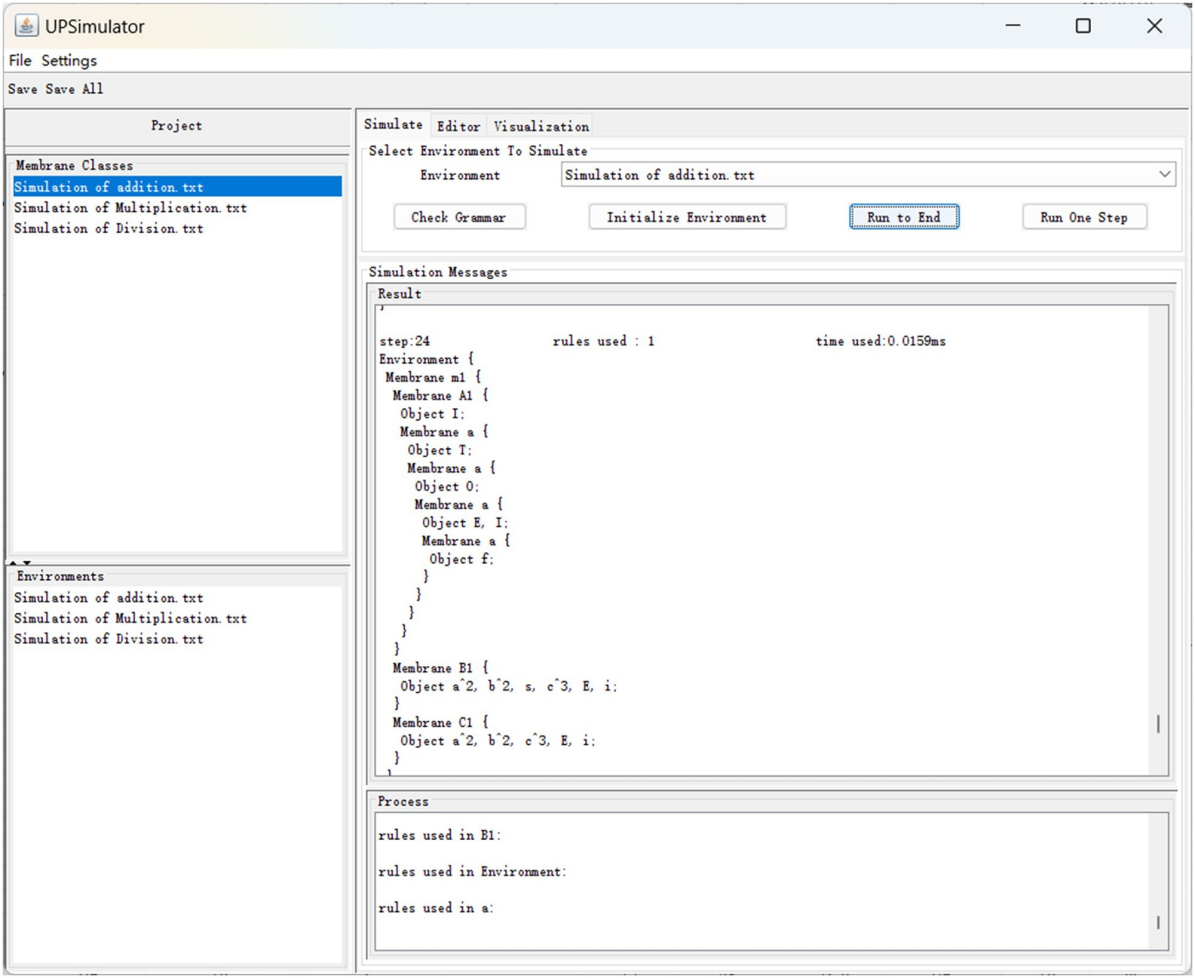
Simulation of *1T00+1T1 = 10T1*.

Rewriting the rules in Section 3.1 in the above format, the result is shown in Fig 11. Objects *I*, *T*, *O*, *I* are retained in membranes *M*_1,…,4_ separately, and a total of 24 time slices are used. It is one time slice more than the actual projection, because object *E* has to enter membrane *C* before releasing the addition in UPS, this will consume one time slice.

### 4.3 Simulation of multiplication

The membrane structure for multiplication is the same as for addition; the membrane class “M” contains the membrane class “Mul”, membrane class “B” and membrane class “C”. Membrane classes B and C are used to input the multiplicand and multiplier into membrane *m*_1_. The overall membrane structure is as follows:


Environment {
    Membrane M m1 {
        Object Y;
        Membrane Mul A1 {
            Object f;
        }
        Membrane B B1 {
            Object A, T, I, s;
        }
        Membrane C C1 {
            Object T, I;
        }
    }
}


**Fig 12:**
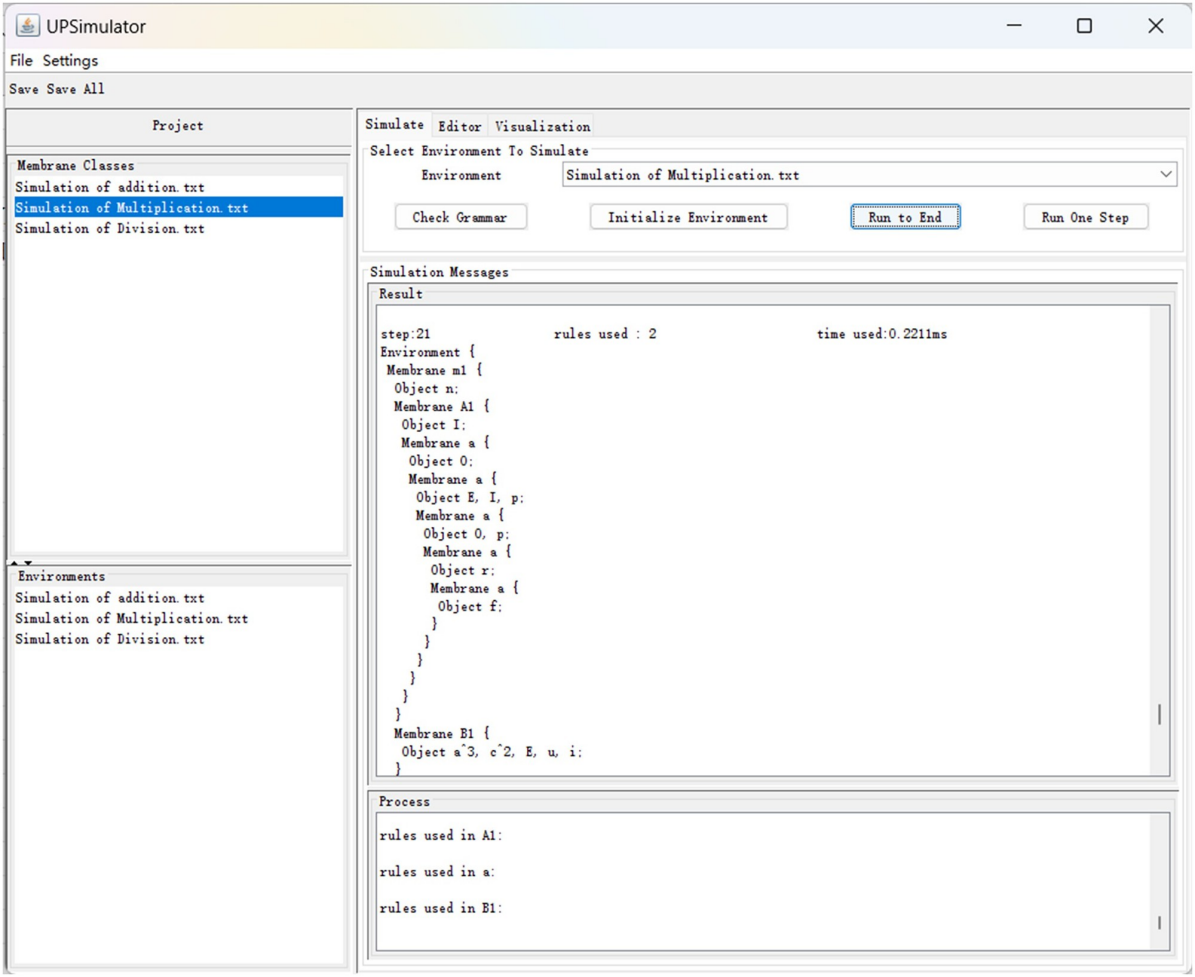
Simulation of 1TT*1T = 101.

Rewriting the rules in Section 3.2 with UPLanguage and running it yields the results as shown in Fig 12. Objects I, O, and I are retained in membranes *M*_1,…,3_. It takes a total of 21 time slices. This duration is one time slice more than the projection because, in the UPS, object E must enter membrane C before releasing the multiplier.

### 4.4 Simulation of division

For the simulation of Π^/^, the rules of Π^/^ are described in UPLanguage. We define a membrane class “M” which contains the membrane class “Div”, membrane class “B” and membrane class “C”. Membrane classes B and C are used to input the dividend and divisor into membrane *m*_1_. The overall membrane structure is as follows:


Environment {
    Membrane M m1 {
        Object Y, K;
        Membrane Div A1 {
            Object f;
        }
        Membrane B B1 {
            Object I, O, C, s;
        }
        Membrane C C1 {
            Object T, I;
        }
    }
}


Division includes the operation of dissolving membranes, and the rules are reformulated in UPLanguage as follows:

- **Rule** *r*_1_: *Ou* → dissolve(*u*, *out*), 1; this rule specifies that when objects *O* and *u* are present together, the membrane is dissolved.
- **Rule** *r*_2_: *v* → (*v*, out)|@*O* & @*C*, 1; this rule indicates that object *v* moves out only in the presence of both objects *O* and *C*.

**Fig 13:**
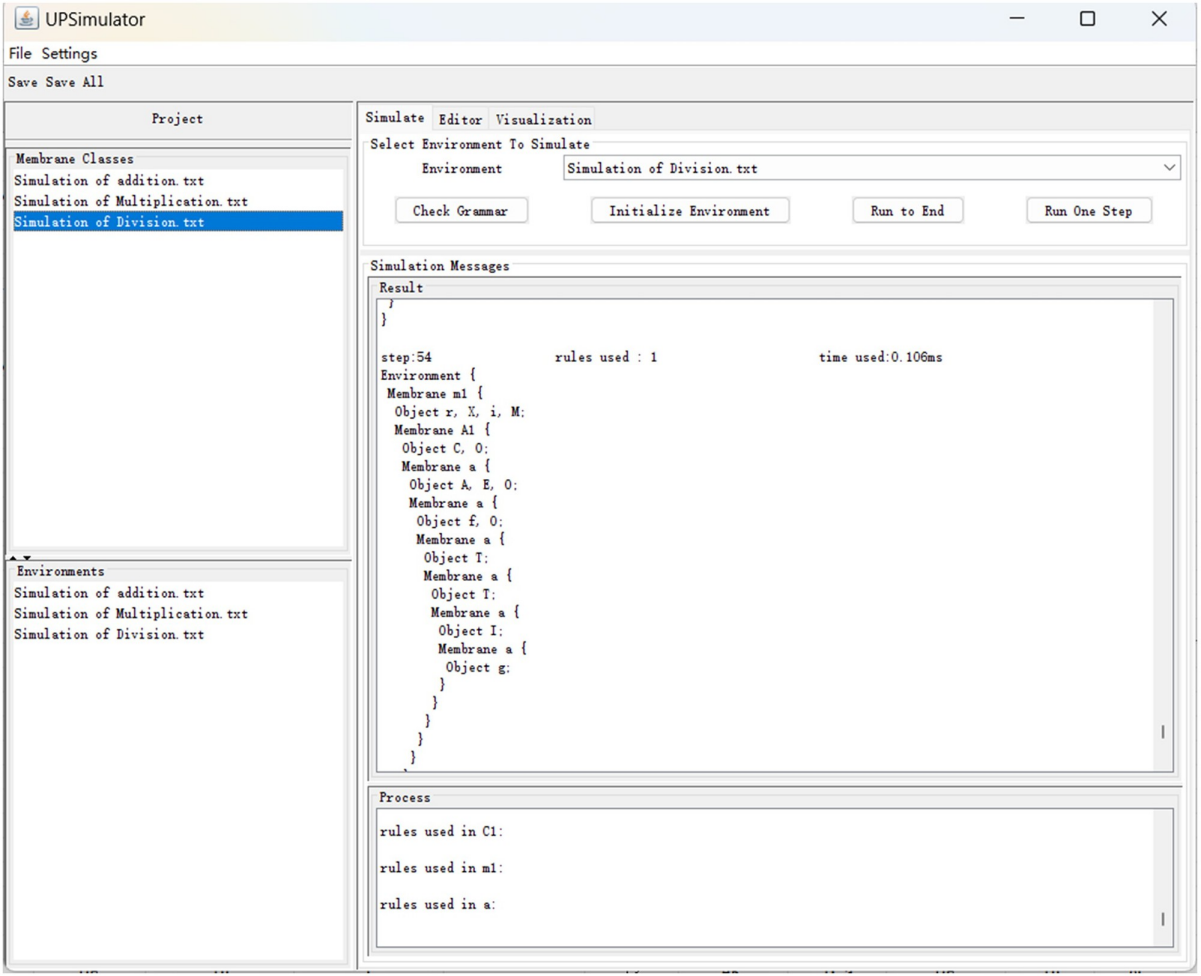
Simulation of *101/1T = 1TT*.

Rewriting the rules in Section 3.3 with UPLanguage and executing the simulation yields the results depicted in Fig 13. The sequence *ITT* is retained in membranes *M*_5_, *M*_6_, *M*_7_ in descending order, consuming a total of 54 time slices. This duration is three time slices longer than the initial projection because Object *E* has to enter membrane *C* before the divisor can be released, taking one additional time slice. Two extra time slices are consumed because, after the dividend and divisor are entered, object *s* is then introduced into membrane 1.

## 5 Conclusion

Membrane computing is characterized by parallelism, distribution, and uncertainty. It has been proved that membrane computing has equivalent computational capabilities with Turing machines, and its powerful parallel computing capability can effectively solve the bottleneck currently faced by electronic computers. The study of arithmetic operation system based on membrane computing has very important academic and practical significance for the realization of a general-purpose bio-computer.

In this paper, a symmetric ternary system is innovatively introduced, which is more adaptable in future bio-computers and can be closer to the natural computation of the human brain than the traditional binary system. A dynamic membrane structure based on membrane computing is designed, which makes the parallel operation of multi-digit numbers possible and improves the computational efficiency. Simulation results show that the designed P-system is not only suitable for basic arithmetic operations, but also can be extended to more complex computational tasks, which provides a new direction for the development of future computing devices.

In the P System we designed, 21 rules are used to implement addition and subtraction, symmetric ternary numbers “*n* + *m*” requires at most 3*n* + 4*m* time slices for addition. 43 rules are used to implement multiplication, “*n* * *m*” require at most 3*n* + 4*m* + 3 time slices for multiplication. And 78 rules are used to implement division, *n*/*m* (*n* ≥ *m*) require at most 3*n* + 5*m* − 2 + 4*i* + (2*m* + 3)*j* time slices, (*i* + *j* = quotient, *i* is the quotient that results when the number of dividend digits is greater than the divisor, and *j* is the quotient that results when the number of dividend digits is equal to the divisor).

Future work will focus on further optimizing the performance of the system and exploring its applicability in more practical application scenarios. Also, we will further investigate their general structure to make it more versatile.
